# Inhibition of the NLRP3 Inflammasome With MCC950 Improves Gut Health in Huntington's Disease Mice

**DOI:** 10.1111/jnc.70419

**Published:** 2026-03-30

**Authors:** Sujan Kumar Sarkar, Millicent N. Ekwudo, Da Lu, Bethany Masson, Pamudika Kiridena, Nicholas van de Garde, Thibault Renoir, James E. Vince, Veerasikku Gopal Deepagan, Anthony J. Hannan, Carolina Gubert

**Affiliations:** ^1^ Florey Institute of Neuroscience and Mental Health Parkville Victoria Australia; ^2^ Florey Department of Neuroscience and Mental Health University of Melbourne Parkville Victoria Australia; ^3^ Department of Anatomy, Histology and Physiology, Faculty of Animal Science and Veterinary Medicine Sher‐e‐Bangla Agricultural University Dhaka Bangladesh; ^4^ The Walter and Eliza Hall Institute of Medical Research Parkville Victoria Australia; ^5^ Department of Medical Biology University of Melbourne Parkville Victoria Australia

**Keywords:** gut health, gut microbiome, gut‐brain axis, Huntington's disease, inflammation, MCC950, NLRP3 inflammasome inhibition

## Abstract

Huntington's disease (HD) is an autosomal dominant neurodegenerative disorder featuring abnormal cognition, psychiatric symptoms, movement, and gastrointestinal function. It is caused by a tandem‐repeat gene mutation encoding an expanded polyglutamine tract in the huntingtin protein. Our group was the first to demonstrate gut microbial disruption in both clinical HD cohorts and validated preclinical models, supporting a role for microbiota–gut–brain axis dysfunction in HD. The NLRP3 inflammasome, a key innate immune sensor that integrates microbial, metabolic, and host‐derived danger signals, has been implicated in HD pathology. However, its contribution to gut health and microbiota‐linked cognitive deficits in HD remains unknown. This study addressed this critical gap by investigating whether targeting NLRP3 can restore gut and brain health in HD through modulation of the microbiota–gut–brain axis. We aimed to investigate the role of the NLRP3 inflammasome in microbiota–gut–brain axis dysfunction by targeting its inhibition. Here, we assessed whether inhibiting NLRP3 can ameliorate cognitive deficits, gut abnormalities, gut microbial alteration, and associated molecular and behavioural disturbances in HD. NLRP3 inflammasome inhibitor MCC950 was administered to R6/1 transgenic HD mice and their wild‐type (WT) littermate controls from 6 to 20 weeks of age. Cognitive and behavioural performance was evaluated using validated tests, alongside assessments of general health and gut function. HD mice exhibited reduced body and brain weight, increased fluid consumption, memory impairments, motor deficits, exacerbated gastrointestinal phenotype, and altered gut microbiota. Treatment with MCC950 did not affect body or brain weight, cognitive and motor performance, and it also did not affect the gut microbial profile of HD mice. However, MCC950 significantly rescued gut health, as evidenced by increased faecal output (in females) and water content (in both males and females), improved stool consistency (in both sexes), and ameliorated macroscopic gut abnormalities. Our findings highlight a promising therapeutic avenue for addressing the significant gastrointestinal anomalies observed in HD. By targeting the NLRP3 inflammasome in R6/1 HD mice, we have identified a novel strategy to improve gut health. These results support further investigation of inflammasome inhibition as a means to alleviate central and peripheral symptoms in HD and improve overall disease management.

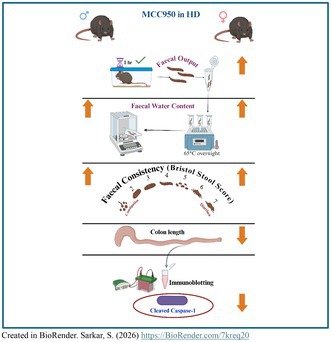

## Graphical abstract

The NLRP3 inflammasome in HD (Huntington's disease) peripheral pathology (in gastrointestinal complications) is vastly unexplored. In this preclinical study, non‐invasive readouts of gut function assessment revealed that inhibiting NLRP3 inflammasome by chronic administration of MCC950 by oral route softened the faecal pellets, improved faecal output and water content in R6/1 HD mice. Cleaved caspase‐1 was also downregulated by the treatment effect of MCC950 in gut tissue. Gastrointestinal dysfunction, including constipation, has been implicated in depression; therefore, these findings identify NLRP3 inhibition as a novel therapeutic approach to ameliorate gastrointestinal complications in HD, with potential to improve patients' gut health and overall wellbeing.

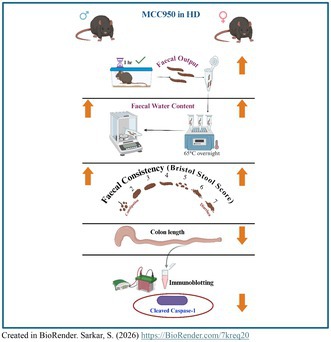

**Abbreviations**
AD: Alzheimer's disease
ASV: amplicon sequence variant
ATP: adenosine triphosphate
bp: base pair
CAG: cytosine‐adenine‐guanine
CAPS: cryopyrin‐associated periodic syndromes
CFC: cued fear conditioning
CLMM: cumulative link mixed model
CS: conditioned stimulus
DNA: deoxyribonucleic acid
EDTA: ethylenediaminetetraacetic acid
F primers: forward primers
FITC: fluorescein isothiocyanate
GI: gastrointestinal
GLM: generalised linear model
GLMM: generalised linear mixed model
GSDMD: gasdermin D
GTT: gut transit time
HD: Huntington's disease
*HTT*
: huntingtin
i.p.: intraperitoneal
IL: interleukin
IP camera: internet protocol camera
LM: linear model
LMM: linear mixed model
MS: multiple sclerosis
MWM: Morris water maze
NE: northeast
NLRP3: NOD‐like receptor protein 3
NOR: novel object recognition
NPI: novel arm preference index
NSFT: novelty‐suppressed feeding test
NW: northwest
PAMPs: pathogen‐associated molecular patterns
PBMCs: peripheral blood mononuclear cells
PBS: phosphate buffered saline
PCA: principal component analysis
R primers: reverse primers
RA: rheumatoid arthritis
RNA: ribonucleic acid
RRID: research resource identifiers
rRNA: ribosomal ribonucleic acid
SE: southeast
SEM: standard error of the mean
SW: southwest
TBI: traumatic brain injury
US: unconditioned stimulus
WT: wild type

## 1 Introduction

Huntington's disease (HD), a currently incurable neurodegenerative hereditary disorder, affects behavioural and psychiatric health, as well as cognitive and motor functions (Huntington's disease—NHS 2021). Indicators of this disease typically manifest between the ages of 40 and 50 (Caldeira Brás et al. 2026). Motor symptoms are often used in diagnosis of HD, but the behavioural and psychiatric problems can appear before that (Parsons and Raymond 2025; Roos 2010). The disease impact in HD extends beyond the patient, affecting the entire family's quality of life (Shaw et al. 2022; Vamos et al. 2007). A tandem‐repeat mutation of the ubiquitously expressed *huntingtin* gene is the root cause of HD pathology (Saudou and Humbert 2016). A striking factor of HD—beyond the neurological psychiatric symptoms—is that patients often present with gastrointestinal (GI) complications like nausea, difficulty swallowing, constipation, diarrhoea, malabsorption, faecal incontinence, among others. GI complications can also contribute to body weight loss (Mehanna and Jankovic 2024). The widely used R6/1 transgenic mouse is an excellent model of HD and ideal for pre‐clinical investigation (Naver et al. 2003). While containing human *HTT* fragments exon1 with approximately 116 CAG (cytosine‐adenine‐guanine) repeat expansion (Mangiarini et al. 1996), this model shows the characteristic symptoms of clinical HD pathology (van Dellen et al. 2000).

Altered gut microbiota profile, known as gut dysbiosis, is evident in HD as demonstrated by preclinical and clinical studies (Kong et al. 2018; Wasser et al. 2020; Du et al. 2021). Gut dysbiosis was associated with impaired weight gain and motor deficits in R6/1 HD mice (Kong et al. 2020). In R6/1 HD mice, gut dysbiosis is also related to changes in the gut microenvironment such as upregulation of faecal water content (Kong et al. 2018). Moreover, gut dysbiosis is found as an early symptomology of these HD mice before the onset of motor abnormalities (Kong et al. 2020). Interestingly, altered gut microbiota in people living with HD has been linked to inflammatory responses, as evidenced by the association of certain genera of intestinal microbes (*Intestinimonas*, *Bilophila*) with the fluctuations of plasma cytokines such as interleukin (IL)‐4 and IL‐6 (Du et al. 2021).

A link has been identified between HD symptoms and peripheral inflammation (Bilal et al. 2024). Inflammasomes are innate immune cytoplasmic multiprotein receptor complexes frequently expressed in macrophages, monocytes, epithelial cells and neutrophils (Honda et al. 2023; Martinon et al. 2007; Schroder and Tschopp 2010). The NLRP3 (NOD‐like receptor protein 3) inflammasome is a key component of the innate immune system that can be activated by danger‐associated signals such as misfolded protein aggregates and environmental microparticles (e.g., uric acid and silica crystals) (Rashidi et al. 2020), extracellular ATP (adenosine triphosphate) and many different microbial molecules (Kelley et al. 2019; Shi et al. 2015; Xu et al. 2024). Once formed, the NLRP3 inflammasome complex triggers a potent inflammatory response through caspase‐1 cleavage and activation of both pore‐forming gasdermin D (GSDMD) resulting in the induction of pyroptotic cell death as well as the inflammatory cytokines, IL‐1β and IL‐18. Pathological NLRP3 signalling has been implicated in a range of inflammatory conditions, including Cryopyrin‐associated periodic syndromes (CAPS), Alzheimer's disease (AD), multiple sclerosis (MS), traumatic brain injury (TBI), rheumatoid arthritis (RA), and atherosclerosis (Blevins et al. 2022). In the context of HD, upregulation of the NLRP3 inflammasome has been observed in peripheral blood mononuclear cells (PBMCs) from individuals carrying the HD gene mutation (Glinsky 2008). Additionally, a prominent histological and immunohistochemical changes were demonstrated in the brains of the R6/2 HD model, with significantly increased expression of caspase‐1 and NLRP3 (Paldino, D'Angelo, Sancesario, and Fusco 2020). Pharmacological inhibition of the NLRP3 inflammasome in R6/2 HD mice resulted in decreased cleaved caspase‐1 in the striatum and alleviated neuropathology, but whether these findings are reproducible in alternative HD preclinical models has not been evaluated (Paldino, D'Angelo, Laurenti, et al. 2020).

The NLRP3 inflammasome also plays an important role in gut health by supporting mucosal immunity and maintaining microbial balance (Lin et al. 2022). Disruption of intestinal homeostasis can occur when the NLRP3 inflammasome becomes dysregulated, promoting sustained inflammatory responses (Lissner and Siegmund 2011). Since pathogen‐associated molecular patterns (PAMPs) like microbial secretory products can activate the inflammasomes (Medzhitov 2007; Mogensen 2009), the gut microbiome represents a critical modulator of NLRP3 inflammasome activity. This relationship is bidirectional, as NLRP3 activation can also influence the composition and function of the gut microbiota, highlighting dynamic crosstalk between host immune signalling and microbial ecology (Zheng et al. 2020; Zmora et al. 2017).

Altered systemic and gastrointestinal inflammatory profiles are evident in HD patients (reviewed in Ekwudo et al. 2024), and changes in colonic cytokines (IL‐7R) have also been observed in HD mouse models (Gubert et al. 2022). Targeting the gut microbiota in HD preclinical models via high fibre diets, prebiotics, and faecal transplants has shown promising therapeutic potential, including improvements in gut health, cognition, and depressive‐like symptoms (Ekwudo et al. 2025; Gubert et al. 2022, 2023). However, it remains unknown whether NLRP3 signalling might contribute to gut pathologies reported in HD.

In this study, we hypothesised that inhibiting NLRP3 would reduce intestinal, cognitive and motor dysfunction that occurs in HD. Unexpectedly, using the R6/1 mouse model of HD, we found that the specific NLRP3 inhibitor, MCC950 (Coll et al. 2015, 2019), did not improve HD defects in cognition and motor performance but did prevent intestinal disease. These findings address a significant gap in our understanding of HD pathology that lacks clinical intervention and identify a novel therapeutic approach that could improve the quality of life for HD patients.

## 2 Methodology

### 2.1 Husbandry and Treatment

R6/1 HD and wild‐type (WT) pups were generated by mating CBA × C57Bl/6 background male R6/1 transgenic mice (originally sourced from The Jackson Laboratory, USA) with female CBA × C57Bl/6 F1 mice. Tail specimens were sampled for DNA genotyping (Gubert et al. 2023). At 5 weeks of age, after genotyping, a total of 82 mice (42 male and 40 females of both WT and HD) were randomly divided. After birth, each mouse was assigned with a 4‐digit unique ID by core animal service of the Florey. Last two digits of ID number were marked by clipped toes. In hind paws, toes were clipped to represent number 1–10 starting from first left toe of left hind paw to last right toe of right hind paw. In forepaw, toes were clipped to represent 20–90 (20, 30, 40, 50, 60, 70, 90) starting from first left toe of left forepaw to last right toe of right forepaw. Mouse ID (last two digits) ranging from 1 to 19 can be identified by checking the clipped toes of hind paw. For example, clipping of toe number 10 and 9 will represent the digit 19, similarly clipping of 2nd and 10th number toe will represent the digit 12 or simply clipping of toe number 5th will represent the ID 05. On the other hand, if any mouse has ID (last two digits) over 20 could be identified by checking the forepaws or combination of both forepaws and hind paws clipped toes. For example, clipping of 20th toe will represent digit 20, while clipping of 20th and 1st will represent the digit 21. After assigning the unique ID, list of mice with genotype and sex was provided to the researcher from core animal service in excel sheet. Researchers were not involved during the ID assignment and toe clipping. To assign the mice in different groups, stratified block randomisation was followed. Mice were stratified by sex and genotype, then mice IDs were randomly picked and assigned to treatment and control groups, keeping 2–3 mice per cage with same sex and same genotype. Mice with same birth date were not kept in same cages.

As the coprophagic nature of mice might influence the gut microbiota profile of cage mates from different groups, we co‐housed 2–3 mice per cage with the same genotype, sex and treatment. Mice had ad libitum access to sterilised food and drinking water and were reared with standard animal husbandry practices with a 12/12 h dark and light cycle and controlled temperature (22°C) and humidity (45%). Cages and bedding were changed once a week. Starting from 6 weeks of age and up to 20 weeks, mice were treated with MCC950 sodium (Selleck Chemicals, Catalogue No. S7809) (delivered in drinking water) versus vehicle (drinking water only) (*n* = 10–11 per group). We supplied ad libitum MCC950 solution to the treated groups and ad libitum standard drinking water (vehicle) to the control groups. The dose of MCC950 was 0.3 mg per ml of drinking water as used previously to block NLRP3 signalling in vivo (Gordon et al. 2018), and the solution was replaced twice a week. The consumption of fluid in grams per gram of mouse per day was calculated by measuring the initial and final weight of the water bottles (Gordon et al. 2018; Ioannou et al. 2023). Food weight was also measured every week and calculated as grams per gram of mouse per day. All practices and methodologies with mice were pre‐approved by the Florey Institute of Neuroscience and Mental Health animal ethics committee with prescribed guidelines (FINMH‐2024‐028).

### 2.2 Experimental Design

**FIGURE 1:**
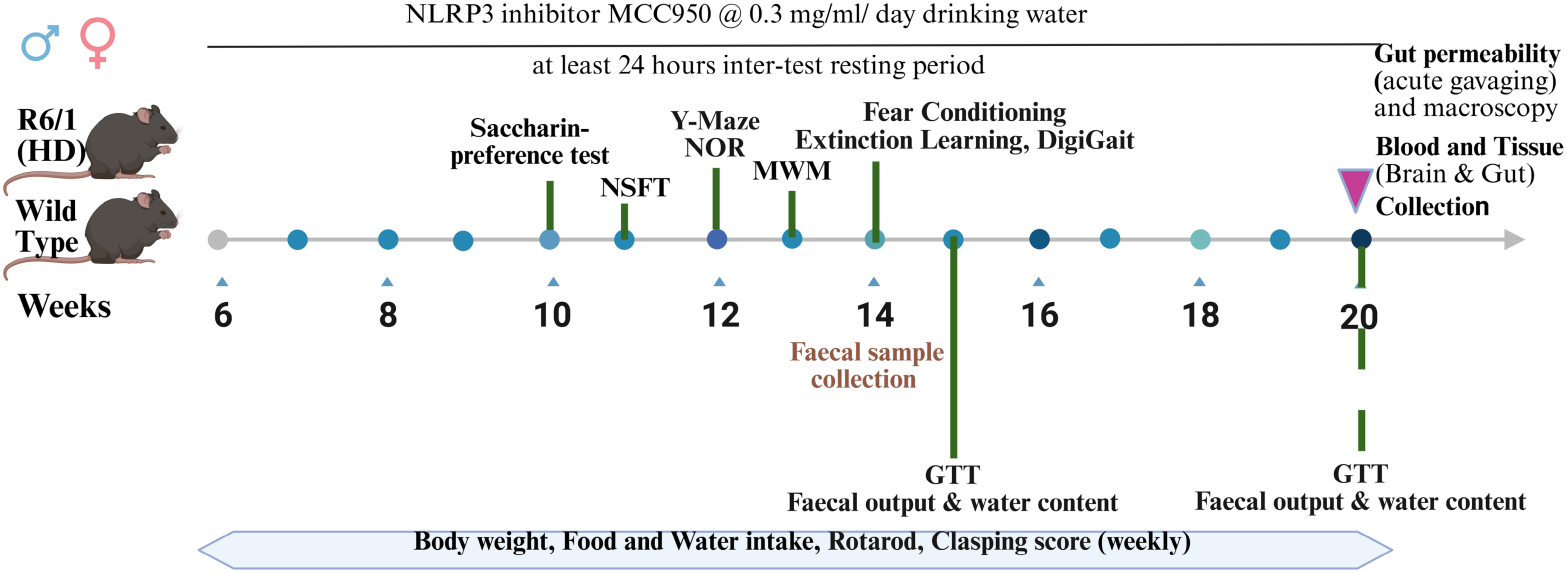
Experimental design. A total of 82 mice (male 42 and female 40) of both HD and WT were treated with MCC950 solution or vehicle (drinking water) from 6 to 20 weeks of age. Depression and anxiety‐like behaviour was evaluated at week 10 and 11. Cognitive function was assessed from 12 to 13 weeks of age. Motor function was evaluated every week by clasping score and rotarpd performance. At week 14, gait was assessed to further evaluate the motor function by digigait analysis. Gut function was evaluated at week 15 and 20 by GTT and faecal output and water content measurement. At week 20, gut permeability and gut macroscopy were assessed. Abbreviations: GTT, gut transit time; HD, Huntington's disease; MWM, Morris water maze; NOR, novel object recognition; NSFT, novelty‐suppressed feeding test; WT, wild type. Figure created in Biorender.com.

The design, including characterising behaviour, motor function, gut health and other measures, is summarised in Figure 1 and extensively described below. Animals were allowed a resting interval of at least 24 h between tests. All tests were performed during the light phase of the day (between 8 AM and 8 PM) except the saccharin preference test which was conducted for 2 days including both light and dark cycle. Faecal samples were collected at 14 weeks of age for microbial profiling. Mice were sacrificed at 20 weeks of age to collect blood and tissue samples. General anaesthesia was induced by an intraperitoneal injection of sodium pentobarbitone at a dose of 80 mg/kg body weight. Pedal withdrawal reflex was monitored to assess the depth of anaesthesia. After confirming the absence of pedal reflex, blood was collected by cardiac puncture. Cervical dislocation was performed immediately after blood collection to ensure death and then, gut and brain samples were collected.

#### 2.2.1 Blinding of Experiment

Experimenter for all tests was blinded about animals' genotypes and treatments during the experimentations. An extra card was used to hide the genotypes' details in each cage; it was maintained throughout the whole experimental period. This extra card only contained the mouse IDs and cage numbers. To blind the treatments, drug solution versus normal drinking water were supplied in identical falcon tubes which were covered in foil paper that did not show any visual difference.

#### 2.2.2 Sample Size Determination

In our 2 × 2 factorial (4 groups) research design, sample size was allocated as 10–11 mice per group based on a power calculation using pwr package in R with effect sizes 0.52–0.55, significance level 0.05, and 80% power (0.80). With this 80% power, the total number of samples could detect biologically relevant differences, considering all the parameters that produced large effect size (Bodden et al. 2022).

Sample size was further cross‐checked using resource equation method as described by published literature (Arifin and Zahiruddin 2017; Charan and Kantharia 2013). In this method, the value of “E” is measured which indicates the degree of freedom (df) of analysis of variance (ANOVA). Any sample size that gives value of “*E*” between 10 and 20 should be sufficient. The formula for calculating the value of “*E*” is as follows:

$$\left[E=\text{number of total animals}-\text{number of groups}\right].$$

In our study, the calculated E value for males was (*E* = 42 − 4) 38 and for females was (*E* = 40 − 4) 36, which were more than 20, indicating the allocated sample size was adequate to detect the biologically relevant differences.

Correspondingly, the minimum and maximum sample requirements per group also highlighted the adequacy of the required number of mice per group. According to Arifin and Zahiruddin (2017), *E* or df is calculated as follows:

$$\mathrm{df}=N-k=\mathrm{kn}-k=k\;\left(n-1\right),$$

where *N* = total sample size, k = number of groups, *n* = sample per group.

After rearranging the formula, n is given as below:

n=df/k+1.

Since our study had four groups, to obtain the minimum and maximum sample size per group based on minimum (10) and maximum (20) requirement of dfs, the formulas are as follows:

$$\text{Minimum}\;n=10/4+1=3.5=4\;\left(\text{round}\right),$$

Maximumn=20/4+1=6.

Hence, the total number of minimum and maximum animals required,

MinimumN=Minimumn×k=4×4=16,

MaximumN=Maximumn×k=6×4=24.

Altogether, in our study sample size per group and total number of samples utilised has met the requirement according to resource equation method calculation as well.

In addition, it was suggested that a sample size of 5 or less could be acceptable for non‐treated control groups in preclinical mouse model studies with established phenotypes (Zhang and Hartmann 2023). In our current study, R6/1 HD mice and their WT counterparts of similar age with well‐established phenotypic characteristics were utilised for the experimentation.

#### 2.2.3 Exclusion and Inclusion of Animals

No animals died during any experiment and no animals were replaced. For the data analysis, all animals were included from all experiments except Digigait, MWM, gut macroscopy, and saccharin preference test. In Digigait analysis, those mice that failed to walk on the running belt during the video recording steps were excluded. Additionally, some recorded videos failed to produce the final processed data due to a technical issue with the readability of video files that led to further exclusion of mice (total 3 males and 2 females). In MWM test, those mice failed to swim or drowned and floated without any movement during the tests were excluded from the analysis (3 males and 4 females). Two males and two females' colon weight and one male and two females' caecal weight were not recorded mistakenly during the culling. During the saccharin preference, those mice cages that were identified with fluid spill during the experimentation were excluded from the analysis (one male and four females).

### 2.3 Body Weight, Water and Food Intake

Fluid and food consumption were recorded weekly along with the body weight. Food and fluid intake were calculated and normalised according to per gram of body weight of the mouse per day (Gubert et al. 2023).

### 2.4 Motor Function Testing

#### 2.4.1 Rotarod Performance Test

We assessed the motor function by evaluating the rotarod (RotaRod, Ugo Basile, Varese, Italy) performance weekly. Week 6 was considered a training period and was kept out of the analysis. The speed of the RotaRod was set to a constant speed of 4 rpm (rotations per minute) and then gradually accelerated to 40 rpm over 300 s. The rotarod test was conducted as described by Pang and colleagues (Pang et al. 2006).

#### 2.4.2 Clasping Score

Clasping behaviour was scored weekly (from week 6 to 20) as an important parameter of motor function in the R6/1 HD mouse. Mice were suspended from the tip of the tail for 30 s, and we scored clasping (0–4) according to the number of paws retracted towards the body (Wright et al. 2015). A score of zero (0) indicates no clasping and no motor abnormalities, while progressively higher clasping (1–4) reflects increased motor dysfunction and incoordination. This test is a subjective assessment, and the same experienced researcher conducted this test each week.

#### 2.4.3 Digigait Analysis

At week 14, to evaluate the gait, we conducted the DigiGait analysis at week 14 using a DigiGait apparatus and DigiGait imaging software (Mouse Specifics Inc., Boston, MA, USA) (Wright et al. 2015). Mouse paws were stained with red food dye, then the animals were placed in a plexiglass chamber on the transparent treadmill belt. Mice were habituated for 1 min. The belt was run at a speed of 15 cm per second. The walking of each mouse on the treadmill was recorded with the video camera connected with DigiGait imaging software for 3–4 s, and then, a set of 6–12 steps (or a minimum of 4) was saved and subsequently analysed using the DigiGait analysis software.

### 2.5 Depression and Anxiety‐Like Behavioural Tests

#### 2.5.1 Saccharin Preference Test

At week 10, we performed the saccharin preference test to evaluate the anhedonia. The experimental protocol was adopted from a previously published study (Mo, Renoir, and Hannan 2014) and modified according to our research design. The test was conducted for 2 days; day 1 was a habituation period, with each mouse single‐housed and provided with two bottles of drinking water (no treatment, no saccharin) and ad libitum food. During day 2, mice were provided one bottle of saccharin solution (0.1%) and one bottle of drinking water (no treatment was added during the period of the test). The side (left or right) of the saccharin solution was randomised. Mice were rehoused in their home cage after testing.

#### 2.5.2 Novelty‐Suppressed Feeding Test

At week 11, we assessed anxiety‐like behaviour in mice by the Novelty‐suppressed feeding test (NSFT) following a previously published protocol (Pang et al. 2009) with some modifications. Mice were food‐deprived for 24 h before the test, maintaining access to MCC950 solution or standard water (ad libitum) according to their assigned treatment groups. During testing, lighting was set to 750 lux, and a 60 cm × 60 cm box was used as the test arena. A small amount of bedding material was taken from each home cage and mixed with new bedding and used in the arena during testing. A single food pellet was attached to a circle of white filter paper and placed in the centre of the box. Body weight was measured before fasting and right before the test. Mice were placed into one of the four corners of the box and allowed to explore the arena and pellet for a maximum of 10 min, and video was recorded using an IP (Internet Protocol) camera. The time when mice started eating the pellet was recorded as the latency to feed. Mice were removed from the arena immediately after starting to bite the pellet, and those mice that did not bite the pellet at all were removed from the arena after 10 min. After the latency test, mice were single‐housed with a pre‐weighed food pellet for 5 min. The pellet consumption was assessed as the difference between the pre‐weighed pellet and the weight after feeding. Males and females were tested on different days.

### 2.6 Cognitive Function Test

#### 2.6.1 Y‐Maze Test

We performed the Y‐maze test to assess the short‐term spatial memory and learning at week 12 according to a published protocol (Mo, Renoir, and Hannan 2014). Total distance travelled during trial 1 was assessed to evaluate any movement‐related impairment in mice. During trial 2, a novel arm preference index (NPI) was calculated as the time spent in the novel arm divided by the average time spent in novel and familiar arms was analysed during the last 4 min of the experiment. Time spent in each arm (novel and familiar) and latency to leave the home arm were also evaluated.

#### 2.6.2 Novel Object Recognition Test

At week 12, the novel object recognition test was performed to assess short‐term memory and learning as previously published (Leger et al. 2013). Novel object recognition and discrimination index were calculated from trial 2, and exploration time for each object was also evaluated.

#### 2.6.3 Morris Water Maze Test

Spatial learning memory was assessed at 13 weeks of age by conducting the Morris water maze (MWM) test over the course of 6 days. Over the first 5 days of the 6‐day protocol, each mouse participated in a training session with 4 trials per day, with the final day as the probe (test) day comprising one trial. A swimming pool with 4 imaginary quadrants filled with opaque, white‐dyed water was used in this test. During the training phases, in a specific quadrant of the pool, a platform was hidden 0.5 cm below the water level. This specific quadrant was considered the target quadrant. Mice were released from different sites (randomised across trials and days) of the pool during each trial and allowed to explore the hidden platform within 60 s. After reaching the platform, mice were left on the platform for 30 s. On probe day (day 6), the hidden platform was removed, and mice were released from a novel starting point (Vorhees and Williams 2006) and allowed to swim for 60 s. We calculated the latency to reach the hidden platform during the 5 training days and during the probe day. Time spent in the target quadrant on the probe day was also evaluated. Relative distance travelled compared to day 1 was also calculated. Additionally, times spent in each quadrant, total distance travelled, and velocity were analysed from day 6 data.

#### 2.6.4 Cued Fear Conditioning and Extinction Learning

To assess the longer‐term associative cognitive function, at 14 weeks of age, we conducted cued fear conditioning (CFC), including fear learning and extinction tests, by measuring the freezing response as a marker of cognitive memory and learning. The inter‐trial period between the conditioning and learning phase was 72 h. For the test, each mouse was placed in a single metallic chamber (Med Associates Inc., Fairfax, VT, USA) with a stainless‐steel rod floor and one of two contextual environments, either a lined or clear background, paper towel or bedding beneath the steel rod floor and lights on or off. During the fear conditioning, a conditioned and unconditioned stimulus were paired. An auditory tone (80 dB, 5000 Hz, 10 s) was set as a conditioned stimulus (CS) while a foot shock (electric shock, 0.6 mA, 1 s) was set as an unconditioned stimulus (US). As a baseline of conditioning, mice were habituated for 2 min while baseline freezing was recorded. After that, mice were exposed to 6 paired CS‐US with a tone of 10 s and a foot shock of 1 s co‐terminating with the tone. The interval between stimulus presentations was set to 110 s. To confirm that the freezing was a conditioned response to the tone, it was analysed during the 9 s before the commencement of the foot shock. After the last tone, mice were allowed to stay in the chamber for 2 min before returning to their home cages. We assessed extinction learning 72 h after conditioning. During the extinction day, CS was evaluated in the alternative environmental setup to avoid the pairing of the chamber environment and tone in the CS. Mice were again habituated for 2 min to measure baseline freezing for extinction learning. Extinction learning involved 45 presentations of the CS, unpaired from the US, with an interval of 10 s between presentations. To evaluate extinction learning, freezing percentage across 9 blocks of five CS presentations was analysed. During the interval between conditioning and extinction, mice were kept undisturbed. Documented freezing responses of both tests were analysed employing the video freeze software package (VideoFreeze, Med Associates Inc.) (Gubert et al. 2022).

### 2.7 Gut Profiling

At 15 and 20 weeks of age, the whole GTT, faecal output and water content, as well as stool consistency (Bristol stool score) were assessed. GTT, faecal output and water content were characterised as previously published (Ekwudo et al. 2025; Gubert et al. 2023). To determine GTT, each animal was gavaged with 6% carmine‐red solution in 0.5% methylcellulose and individually housed for 9 h (with chow and fluid provided ad libitum). While watching for the red pellet, the faeces were scored using the Bristol stool scale to determine the consistency. For faecal output and water content, mice were single‐housed for 1 h, and the number of pellets excreted was counted and collected in a pre‐weighed Eppendorf tube at 15‐min intervals; then the water content of the faeces was determined with the gravimetric approach.

Furthermore, to probe intestinal permeability at the age of 20 weeks, mice were food‐deprived overnight and gavaged with 0.15 mL of 4 kDa fluorescein isothiocyanate (FITC) dextran (100 mg FITC per ml of filtered phosphate‐buffered saline). After 4 h post‐gavage, 0.2–0.5 mL blood was collected through cardiac puncture using a 1 mL syringe with a 30 G needle following anaesthesia procedure as described in Section 2.2. Blood was immediately transferred to a tube containing anticoagulant (EDTA‐ethylenediaminetetraacetic acid), and then plasma was isolated after centrifugation at 5000 rpm for 15 min. The fluorescence intensity of FITC in the plasma was quantified according to a published protocol (Kong et al. 2023). Fluorescence intensity was measured by using the microplate reader PHERAstar FSX. The excitation was set at 485 nm, while the emission wavelength was set at 528 nm.

Additionally, we assessed the gut macroscopy, including the length and weight of the colon and cecum, as previously described (Gubert et al. 2021; Kong et al. 2023).

### 2.8 Gut Microbiome Profiling

#### 2.8.1 Faecal DNA Extraction and 16S rRNA Sequencing

Faecal samples were collected at 14 weeks of age for 16S rRNA gene sequencing analysis. Mice were single‐housed in clean cages with lids for faecal pellet collection. Fresh faecal samples (2–3 pellets per mouse) were collected in Eppendorf PCR tubes and immediately placed on dry ice, then stored at −80°C until further analysis (up to 6 months without freeze and thaw cycle) (Gubert et al. 2023).

Microbial DNA was extracted from the faecal samples and 16S rRNA gene amplicon sequencing was conducted in line with the Earth Microbiome Project (https://earthmicrobiome.ucsd.edu/protocols‐and‐standards/16s/) (Gubert et al. 2023). The PowerSoil HTP kit (Qiagen) was used to extract genomic DNA from faecal samples. Then, prokaryotic 515F and 806R primers (targeting the V4 hypervariable region of the 16S rRNA gene) were used to amplify the genomic DNA. V4 16S rRNA gene sequences amplicons were created using paired‐end 150 bp and sequenced on the Illumina MiSeq platform (Gubert et al. 2023).

#### 2.8.2 Bioinformatics Analysis and Statistical Analysis for 16S rRNA Sequencing

Raw amplicon data were denoised using Deblur. All samples passed quality control and were retained for downstream analyses. Taxonomy classification was conducted against the SILVA 138 SSU NR 99 database. For alpha diversity, samples were rarefied to 10 000 reads before performing the calculation for Shannon diversity and a 3‐way ANOVA (genotype × treatment × sex) was used to assess significance. If main effects were detected as significant, a pairwise Wilcoxon test (*p* < 0.05, FDR‐corrected for multiple comparisons) was executed to assess significant differences between groups.

Aitchison distance was calculated for beta diversity analysis, and PERMANOVA with 999 permutations was used to identify group differences among genotype or treatment. When significant main effects were identified, a pairwise PERMANOVA test was conducted to evaluate significant group differences. In both cases, the metrics were computed using the *vegan* R package (Gubert et al. 2023, 2025).

### 2.9 Western Blot Analysis

#### 2.9.1 Tissue Lysis

After culling, mice brain (hippocampus, striatum) and gut (colon) samples were collected and immediately snap frozen on dry ice and then stored in −80 freezer. For the protein extraction, after weighing, the tissue was placed in a 2 mL Eppendorf tube where disc lysis buffer containing 20 mM Tris–HCL pH 7.5, 150 mM NaCl, 2 mM EDTA, 1% Triton X‐100, 10% Glycerol, H_2_O, protease inhibitor cocktail tablet (Roche 4693132001), phospho‐STOP phosphatase inhibitor tablet (Roche 4906837001) was added at 10 μL/mg of tissue. Metal beads (1–2 per sample) were added in the tubes and then samples were homogenised using TissueLyser 85300 in 1‐ to 2‐min bursts, keeping samples on ice in between bursts. After homogenisation, samples were kept on ice for 20 min and then centrifuged at 15 000 rpm keeping the temperature 4°C for 15 min. Supernatant was collected and BCA (Bicinchoninic Acid) assay (Thermo Fisher Scientific, 23225) was performed to quantify the protein concentration. After the BCA assay, samples were diluted with disc buffer and 20% of 5× SDS reducing sample buffer aiming to at 2 μg/μL of protein in the final concentration.

#### 2.9.2 Immunoblotting

Protein samples containing 5× SDS reducing sample buffer were heated for 5–10 min on the heat block at 95°C temperature. After that, samples were cooled at room temperature and then a quick spin was performed. 20 μl sample/well was loaded in 20‐well 4%–12% gradient Bis‐Tris SDS‐PAGE gel (Invitrogen, Cat#NP0321) and the gel was run at 90 V for 10 min and 120 V for 90 min. Proteins were transferred onto nitrocellulose membranes (Amersham, Cat# GE10600073) overnight. Ponceau S was performed to cross check the loading quality of protein. Membranes were blocked for 1 h at room temperature in 5% skim milk (w/v) prepared in tris buffered saline (TBS) containing 0.1% Tween‐20 (TBS‐T). After blocking, membranes were incubated with primary antibodies diluted (1:1000) in 5% skim milk in TBS‐T or 5% BSA at 4°C overnight. Primary antibodies used in the experiment include NLRP3 (Adipogen, Cat# AG‐20B‐0014‐C100), IL‐1β (R & D, Cat# AF‐401‐NA), Caspase‐1 (Abcam, Cat# ab179515).

After primary antibody incubation, membranes were washed in TBS‐T 3 × 5 min and then incubated with relevant horseradish peroxidase (HRP)–conjugated secondary antibodies diluted in 5% skim milk (1:10 000) at room temperature for 1 h. Horseradish peroxidase (HRP)–conjugated secondary antibodies (anti‐mouse secondary, Southern Biotech, Cat# 1010‐05; anti‐goat secondary, Invitrogen, Cat# A16005; anti‐rabbit secondary, Southern Biotech, Cat# 4010‐05). Membranes were washed in TBS‐T 3 × 5 min and then images were captured in Chemidoc Touch Imaging System (Bio‐Rad) after developing the blot with enhanced chemiluminescence (ECL) (Millipore, Bio‐Rad, WBLUF0500).

#### 2.9.3 Quantification and Normalisation Process

Quantification and normalisation were performed followed by the guideline described in the published literature (Pillai‐Kastoori et al. 2020) with modification. Protein quantification was conducted by using Bio‐Rad Image Lab 6.1 Software. Target protein band was measured from the raw chemiluminescence pictures for cleaved caspase‐1 (p10/12). Total protein was quantified from the colorimetric image that was captured after ponceau staining. The lane normalisation factor was then calculated for each lane, using combined total protein signal values for each lane. This factor was calculated by dividing the signal intensity observed for the control (reference lane‐WT control‐first lane) with the signal intensity value for each experimental lane. To apply the normalisation factors, the signal intensity of the target band in each lane was multiplied by the lane normalisation factor for that lane. This process generates the normalised signal intensity value for each sample.

$$\text{From total protein}\;\left(\text{colorimetric image}\right),\text{Normalisation Factor}\;\left(\mathrm{NF}\right)=\text{Signal of reference control}/\text{signal for each lane}.$$

$$\text{From target protein}\;\left(\mathrm{raw}\;\text{chemiluminescence image}\right),\text{Normalised signal}=\mathrm{NF}\times \text{target band intensity}.$$

Fold change=normalised target signal/mean of normalised target signal ofWTcontrol samples.

Then, the normalised target signal for each sample was divided by the mean of normalised target signal observed in the WT control samples. These ratios express the abundance of the target protein as a fold or percentage change, relative to the mean of WT controls.

### 2.10 General Statistical Analysis

Data were visualised using GraphPad Prism version 10.4.2 (633) and displayed as mean ± SEM (Standard Error of the Mean) (*n* = 10–11). Data from males and females were analysed separately due to the sexual dimorphism in HD mice. Statistical analyses were performed in R (version 4.3.3) and R Studio (version 2025.05.1 + 513). We fitted the linear mixed models (LMMs) to conduct the statistical assessment. Genotype and treatment were set as fixed factors and “Cage ID” as a random factor during the analysis of single time point data. Cage effects were considered due to coprophagic nature of mice sharing gut microbiota with their cage mates. For multiple time point data, genotype, treatment and age were set as fixed factors while “Mouse ID” was set as a random factor. Box plots of residuals were evaluated to test the potential outliers. Raw data were verified and corrected if any data entry errors were responsible for the outliers. For analysis, no data points were excluded even though any detected outlier was present as they appeared to be valid observation. Normality and homoscedasticity of residual plots were evaluated to check the assumptions of LMMs were met. Where assumptions were violated, we ran GLMM (generalised linear mixed model)/GLMM repeated measure analyses. Where “Cage ID/Mouse ID” did not contribute as a random factor, we simplified the model and ran a linear model (LM) or GLM (generalised linear model) without the random factor. CLMM (Cumulative Link Mixed Model) was used to analyse the clasping scores and stool consistency. Post hoc pairwise comparisons of the interaction effect were performed using the emmeans package in R, followed by a Bonferroni adjustment. Main effects and interaction effects were evaluated with significance set to the *p*‐value of *α* < 0.05.

## 3 Results

### 3.1 MCC950 Did Not Improve HD Phenotypic Characteristics

**FIGURE 2:**
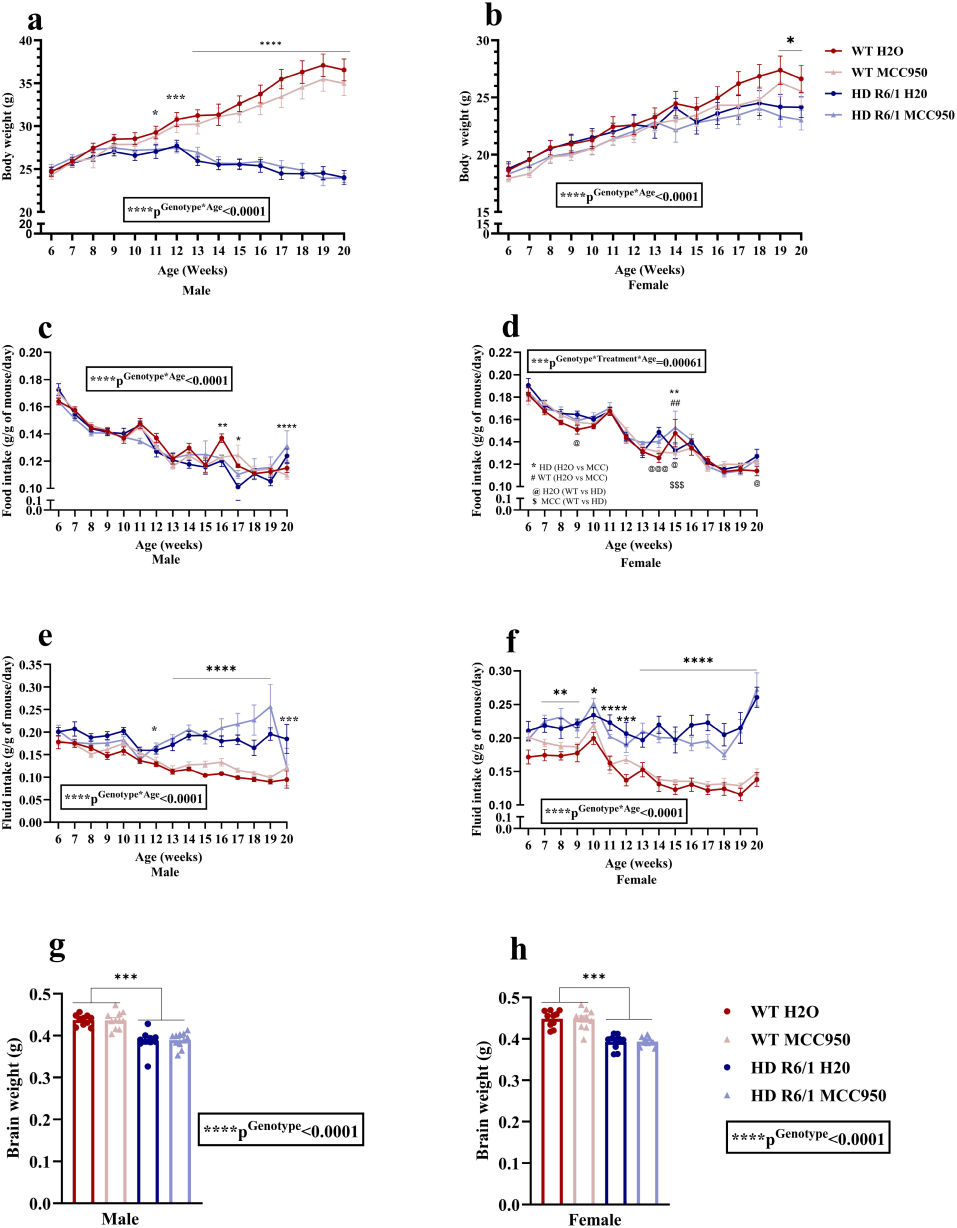
Effects of MCC950 on phenotypic features of HD and WT littermate control mice. Body weight (a, b), food intake (c, d), fluid intake (e, f), brain weight (g, h). Data are displayed as mean ± SEM. Statistical analyses were performed using linear mixed model repeated measure analysis/generalised LMM repeated measure analysis (a–f) and linear mixed model (LMM)/generalised LMM (g–h) followed by Bonferroni post hoc adjustment with emmeans package in *R*. *p* value (*α*) = 0.05. ****p < 0.0001, ***p < 0.001, **p < 0.01, *p < 0.05. *n* = 10–11 (male), *n* = 10 (female). Symbols in Figure 2d, indicate the significance of the post hoc comparisons as follows: *HD (H2O vs MCC), #WT (H2O vs MCC), @H2O (WT vs HD), $MCC (WT vs HD) and for each comparsion one to four symbols in the graph represent *p* < 0.05 to *p* < 0.0001, respectively. HD, Huntington's disease; MCC, MCC950; WT, wild type.

R6/1 HD and WT mice were provided with MCC950 from 6 weeks of age (Figure 1). Body weight, food and fluid consumption were measured weekly. A significant interaction between genotype and age (*F*_(14,532)_ = 117.75, *p* ^Genotype×Age^ < 0.0001) was observed in both male (Figure 2a) and female (*χ* ^2^(14) = 127.80, *p* ^Genotype×Age^ < 0.0001) (Figure 2b). Post hoc analysis showed that HD male mice had a decreased body weight from week 11 to week 20, while female HD mice showed decreased body weight at 19 and 20 weeks of age compared to their WT littermates.

With the food intake, there was a significant interaction effect of genotype and age in males (*F*_(14,532)_ = 3.93, *p* ^Genotype×Age^ < 0.0001) and interaction of genotype, treatment and age in females (*F*_(14,504)_ = 2.75, *p* ^Genotype×Treatment×Age^ = 0.00061) (Figure 2c,d). According to post hoc analysis, male HD mice at 16 and 17 weeks of age consumed less food, but at 20 weeks of age consumed more food per g of body weight than the WT mice. Post hoc tests in females showed that HD control mice consumed more food at week 9, 14 and 20 and less food at 15 weeks of age than the WT control mice. We also observed that MCC950‐treated female HD mice consumed more food than the MCC950‐treated female WT mice with the same treatment at 15 weeks of age. However, compared to the female control groups, MCC950 treatment showed a significant difference in HD and WT mice, indicating an increased food intake in HD mice, while a decreasing trend in WT females at 15 weeks of age.

Regarding fluid intake, we found a significant interaction effect of genotype and age for both males (*χ* ^2^(14) = 119.01, *p* ^Genotype×Age^ < 0.0001) and females (*χ* ^2^(14) = 225.44, *p* ^Genotype×Age^ < 0.0001) (Figure 2e,f). Post hoc analysis revealed that male HD mice consumed significantly more fluid than WT mice from 12 to 20 weeks of age. In females, post hoc analysis demonstrated that starting from 7 weeks, HD mice drank more water until the end of the experiment (Figure 2f). Next, we assessed the brain weight at 20 weeks and found a significant effect of genotype in both males (*F*_(1,15.821)_ = 50.1660, *p* ^Genotype^ < 0.0001) and females (*χ* ^2^(1) = 46.1645, *p* ^Genotype^ < 0.0001) where HD mice displayed significantly lower brain weights than the WT mice (Figure 2g,h).

### 3.2 MCC950 Did Not Improve HD Motor Function

**FIGURE 3:**
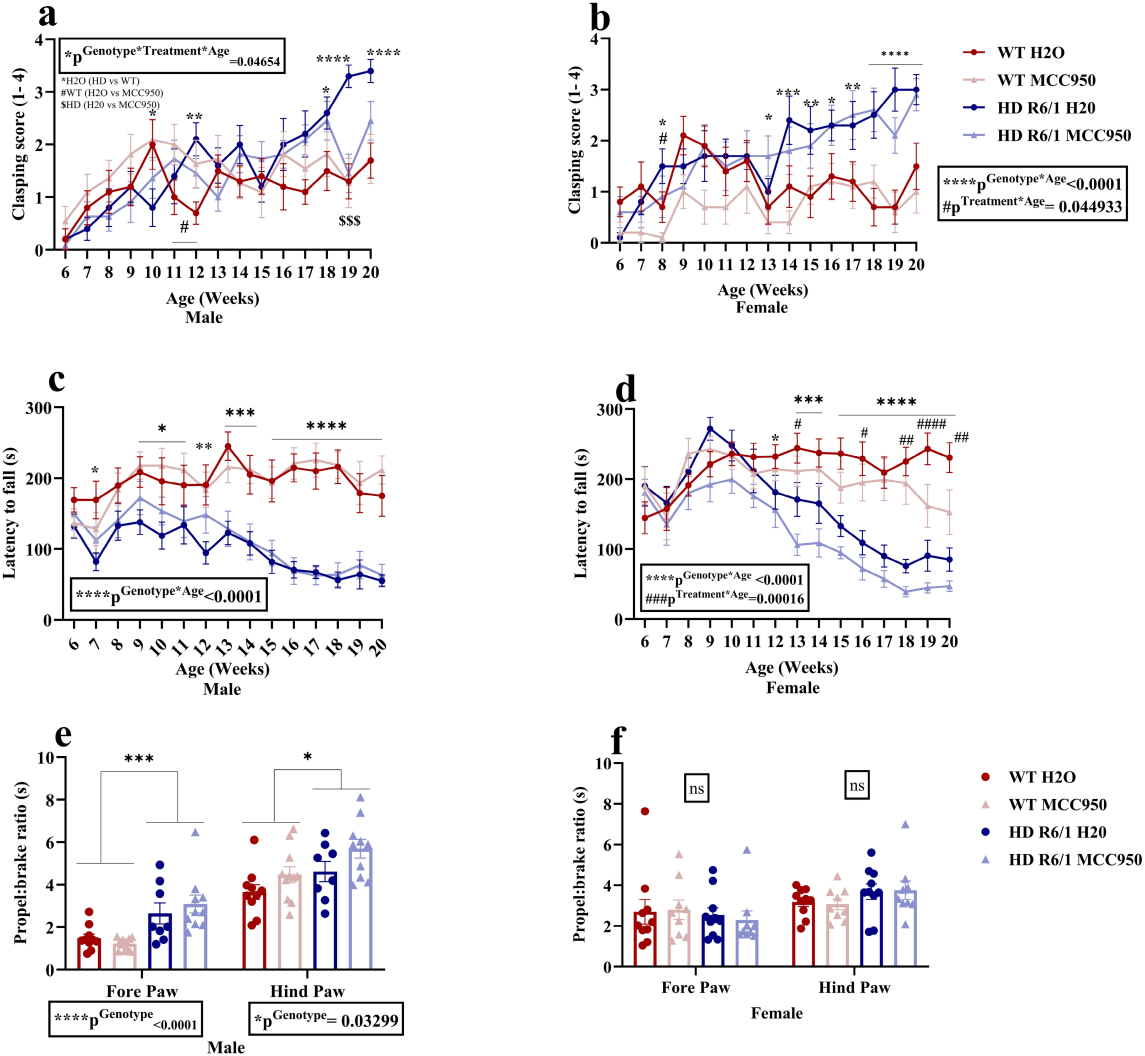
Effects of MCC950 on motor function of HD and WT littermate control. Clasping score (a, b), rotarod performance (c, d), digigait (e, f). Data are displayed as mean ± SEM. Statistical analyses were performed using CLMMs (cumulative link mixed models) (a‐b), linear mixed model repeated measure analysis/generalised LMM repeated measure analysis (c, d), and linear mixed model (LMM)/generalised LMM (e, f) followed by Bonferroni post hoc adjustment with emmeans package in R. *p* value (*α*) = 0.05 and ****p < 0.0001, ***p < 0.001, **p < 0.01, *p < 0.05, ns= non‐significant *n* = 10–11 (male), *n* = 10 (female) for clasping score and rotarod; *n* = 8–10 (male), *n* = 9–10 (female) for digigait. Symbols in Figure 3a, indicate the significance of post hoc comparisons as follows: *H2O (HD vs WT), #WT (H2O vs MCC950), $HD (H2O vs MCC950). Also, in Figure 3b and 3d, # symbol represents the significant interaction between treatment and age. One to four symbols in the graph represent *p* < 0.05 to *p* < 0.0001, respectively. HD, Huntington's disease; WT, wild type.

We evaluated the motor function every week by assessing the clasping score and rotarod performance. In the male, we found a significant interaction between genotype, treatment and age (*χ* ^2^(14) = 23.9438, *p* ^Genotype×Treatment×Age^ = 0.04654) in clasping scores (Figure 3a). Post hoc analysis showed a significant difference between control HD and WT mice during 12, 18–20 weeks of age, indicating HD mice displayed a higher clasping score. In females, interaction between genotype and age was also significant (*χ* ^2^(14) = 27.52, *p* ^Genotype×Age^ < 0.0001) (Figure 3b). Post hoc analysis demonstrated that HD mice had a lower score a 8 weeks of age, before scoring higher than WT mice from 13 to 20 weeks of age. Additionally, female mice showed an interaction of treatment and age (*χ* ^2^(14) = 24.0704, *p* ^Treatment×Age^ = 0.044933) and post hoc analysis revealed a significant difference between MCC950 and vehicle control only at 8 weeks of age (Figure 3b). In rotarod test, we found a significant interaction effect of genotype and age in both males (*χ* ^2^(14) = 183.50, *p* ^Genotype×Age^ < 0.0001) (Figure 3c) and females (*χ* ^2^(14) = 228.00, *p* ^Genotype×Age^ < 0.0001) (Figure 3d) where HD mice had shorter latency to fall (males from 7 and 9–20 weeks, females from 12 to 20 weeks) than the WT mice. In females, a significant interaction between genotype and treatment was also found (*χ* ^2^(14) = 41.29, *p* ^Genotype×Treatment^ = 0.00016) (Figure 3d) and post hoc test showed that MCC950‐treated mice had decreased performance than the water‐treated groups at 13, 16, and 18–20 weeks of age. To further assess the motor function, we evaluated the gait performance at 15 weeks of age. We found a significantly higher propel to brake ratio in both fore limbs (*χ* ^2^(1) = 17.1775, *p* ^Genotype^ < 0.0001) and hind limbs (*F*_(1,15.819)_ = 5.4572, *p* ^Genotype^ = 0.03299) of male HD mice than their WT littermates (Figure 3e). Compared to the vehicle‐treated groups, MCC950 groups did not show any significant difference in the propel:brake ratio in either of the paws of male mice (Figure 3e). In females, there was no significant variation between groups when the propel to brake ratio of fore and hind limbs was assessed. Other parameters of swing time, stance time, stride time and stride length showed no genotype or treatment effect in either sex (Figure S1a–d). For absolute paw angle, male mice in general showed no signifcant difference, while female HD mice exhibited lower absolute paw angle (hind paw only) (*F*_(1,34)_ = 6.8512, *p* ^Genotype^ = 0.01313) compared to WT mice (Figure S1e).

### 3.3 MCC950 Did Not Improve HD Depression and Anxiety‐Like Behaviours

Anhedonia (a key indicator of depression‐like behaviour) was assessed by conducting a saccharin‐preference test at week 10. In this experiment, no effects of genotype or treatment were identified in either sex (Figure S2a,b). To measure anxiety‐like behaviour, we conducted the novelty suppressed feeding test at 11 weeks of age. In the fasting component of the test, we found that HD males displayed lower overnight body weight loss when compared to the WT mice (*F*_(1,15.981)_ = 7.6906, *p* ^Genotype^ = 0.01358) (Figure S2c). In females, the body weight loss did not differ in any groups (Figure S2d). Additionally, the latency to start feeding and the amount of food consumed post‐test did not differ across groups, regardless of sex (Figure S2e–l). Albeit there was a marginal effect of genotype (*F*_(1,15.748)_ = 4.2661, *p* ^Genotype^ = 0.05574), when food consumption was measured in ratio with final body weight (after fasting) in males (Figure S2f), suggesting HD mice consumed more food than the WT mice. As some of the mice did not bite the pellet during the entire period of the latency test (10 min), latency performance was further analysed with Kaplan–Meier survival curves analysis (Figure S2i–l). The survival probability test showed no significant difference between groups for both males (*p* = 0.426) and females (*p* = 0.0551).

### 3.4 MCC950 Did Not Improve HD Short‐Term Memory and Cognition

**FIGURE 4:**
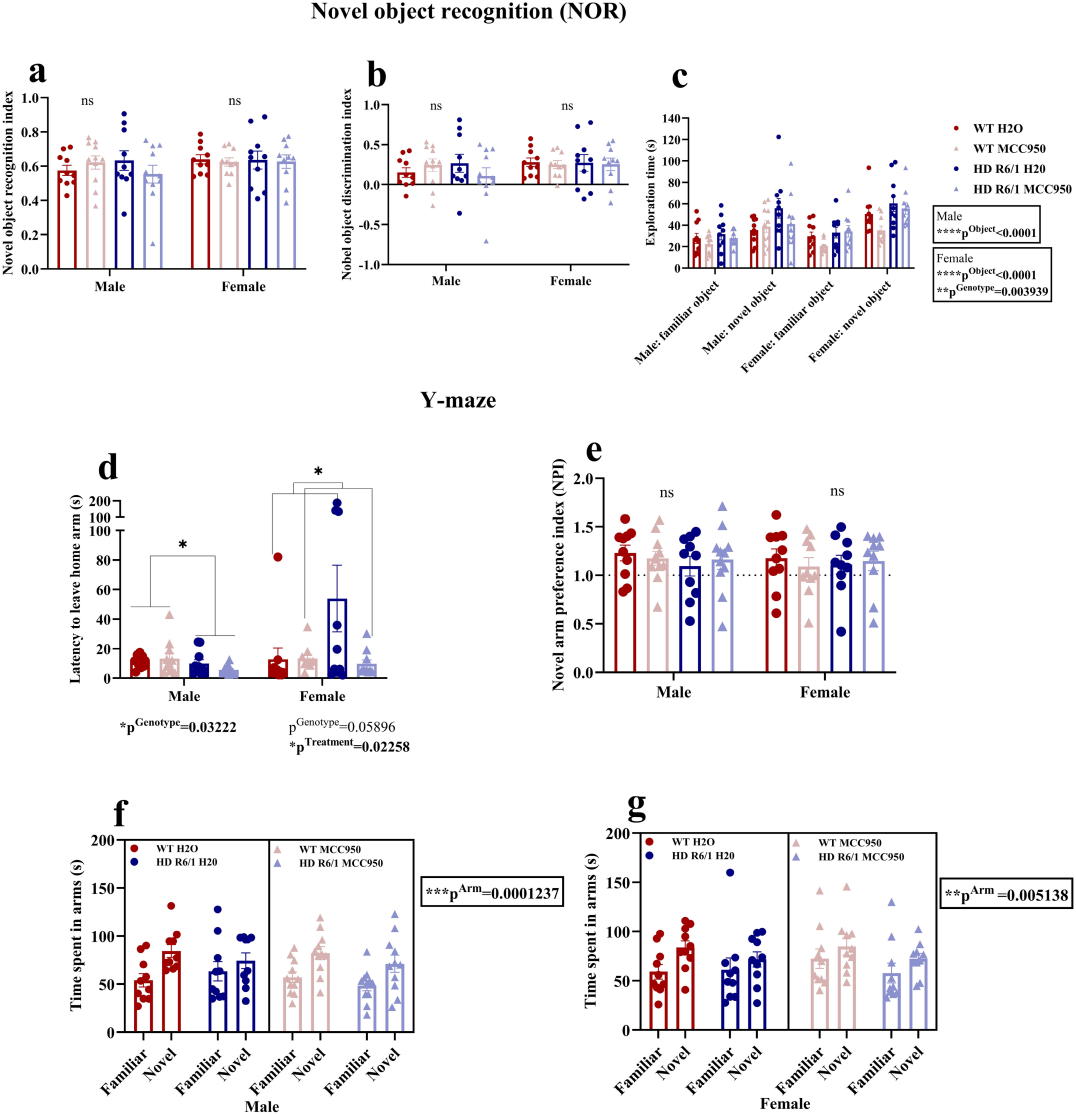
Effects of MCC950 on short‐term memory and learning of HD and WT littermate control mice. Novel object recognition (NOR) test (a–c), Y‐maze test (d–g). Data are displayed as mean ± SEM. Statistical analyses were performed using linear mixed model (LMM)/generalised LMM (a–c), generalised linear model (d) and linear mixed model/generalised LMM repeated measure analysis (c, f, g) followed by Bonferroni post hoc adjustment with emmeans package in R. *p* value (*α*) = 0.05 and ****p < 0.0001, ***p < 0.001, **p < 0.01, *p < 0.05, ns = non‐significant. *n* = 10–11 (male), *n* = 10 (female). HD, Huntington's disease; WT, wild type.

To assess short‐term memory and learning, at week 12, the Y‐maze test and NOR test were performed. In NOR, after analysing the novel object recognition index and discrimination index, we found that the genotypes and treatment groups did not differ significantly (Figure 4a,b) for either sex. Regarding exploration time of familiar and novel objects (Figure 4c), both male (*F*_(1,38)_ = 18.9540, *p* ^Object^ < 0.0001) and female (*χ* ^2^(1) = 69.7722, *p* ^Object^ < 0.0001) mice showed a significant main effect of object. In females, there was a significant effect of genotype (*χ* ^2^(1) = 8.3118, *p* ^Genotype^ = 0.003939).

During the 10‐min trial of the Y‐maze test, when total distance travelled was assessed to evaluate the locomotory activity, no significant difference between groups was observed (Figure S1f,g). We evaluated the latency to leave the home arm during the second trial and found a significant genotype effect (*F*_(1,38)_ = 4.9428, *p* ^Genotype^ = 0.03222) in males (Figure 4d), indicating that HD mice spend less time leaving the home arm, unlike the WT mice. In female mice, a trend of genotype effect was observed (*F*_(1,36)_ = 3.8038, p^Genotype^ = 0.05896) suggesting HD females took longer to leave the home arm while the treatment effect was significant (*F*_(1,36)_ = 5.6775, *p* ^Treatment^ = 0.02258), suggesting that MCC950‐treated mice required a shorter time to leave the home arm (Figure 4d). Regarding the novel arm preference index (NPI), there was no significant difference between groups (Figure 4e). Investigating the time spent in each arm by a mouse, we noticed a significant main effect of arms (novel and familiar) (*F*_(1,38)_ = 18.2798, *p* ^Arm^ = 0.0001237) in males (Figure 4f) and in females (*χ* ^2^(1) = 7.8301, *p* ^Arm^ = 0.005138) (Figure 4g).

### 3.5 MCC950 Did Not Improve HD Long‐Term Spatial Memory and Cognition

To assess the longer‐term associative cognitive function, we conducted cued fear conditioning (CFC), including fear conditioning and extinction at 14 weeks of age. Freezing time in percentage was analysed during both conditioning and extinction trials to assess the memory formation impairment.

**FIGURE 5:**
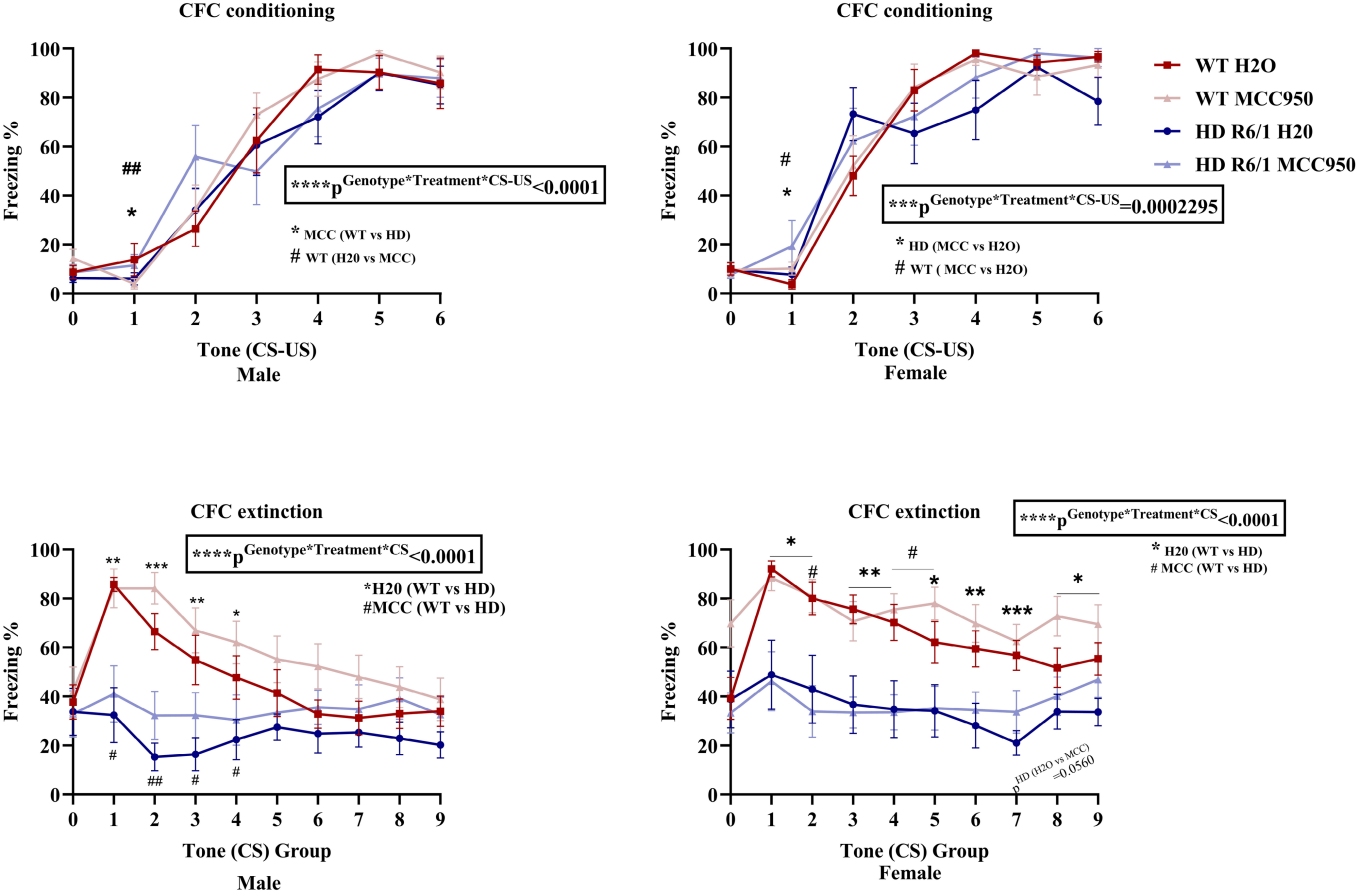
Effects of MCC950 on longer‐term associative cognition and spatial memory of HD and WT littermate control mice (CFC test). Fear conditioning (a, b) and fear extinction (c, d). Data are displayed as mean ± SEM. Statistical analyses were performed using linear mixed model repeated measure analysis/generalised LMM repeated measure analysis (a–d) followed by Bonferroni post hoc adjustment with emmeans package in R. *p*‐value (*α*) = 0.05 and ####,****p < 0.0001, ###,***p < 0.001, ##,**p < 0.01, #,*p < 0.05. *n* = 10–11 (male), *n* = 10 (female). HD, Huntington's disease; MCC, MCC950; WT, wild type.

We did not detect any significant difference in the freezing response at the baseline phase of both conditioning and extinction. During the conditioning phase, we found a significant interaction of genotype, treatment and CS‐US in both males (*χ* ^2^(6) = 94.4135, *p* ^Genotype×Treatment×CS‐US^ < 0.0001) and females (*χ* ^2^(6) = 25.9297, *p* ^Genotype×Treatment×CS‐US^ = 0.002295) (Figure 5a,b). Post hoc tests revealed that in male mice, MCC950‐treated WT mice showed lower freezing than the water‐treated WT mice and MCC950‐treated HD mice during the first stimulus presentation. Female MCC950‐treated mice showed a higher freezing percentage than the water‐treated group. When we analysed the extinction phase, we observed a three‐way interaction between genotype, treatment and CS group in males (*χ* ^2^(9) = 51.3986, *p* ^Genotype×Treatment×CS^ < 0.0001) and females (*χ* ^2^(9) = 75.1628, *p* ^Genotype×Treatment×CS^ < 0.0001) (Figure 5c,d). Post hoc analysis showed that male HD mice froze less regardless of treatment and relative to WT counterparts over CS groups 1 to 4 (Figure 5c). Female HD mice showed lower freezing percentage compared to the WT mice from CS groups 1 to 9 (water‐treated mice) and during CS groups 2, 4, 5 (MCC950 treated‐mice) (Figure 5d).

**FIGURE 6:**
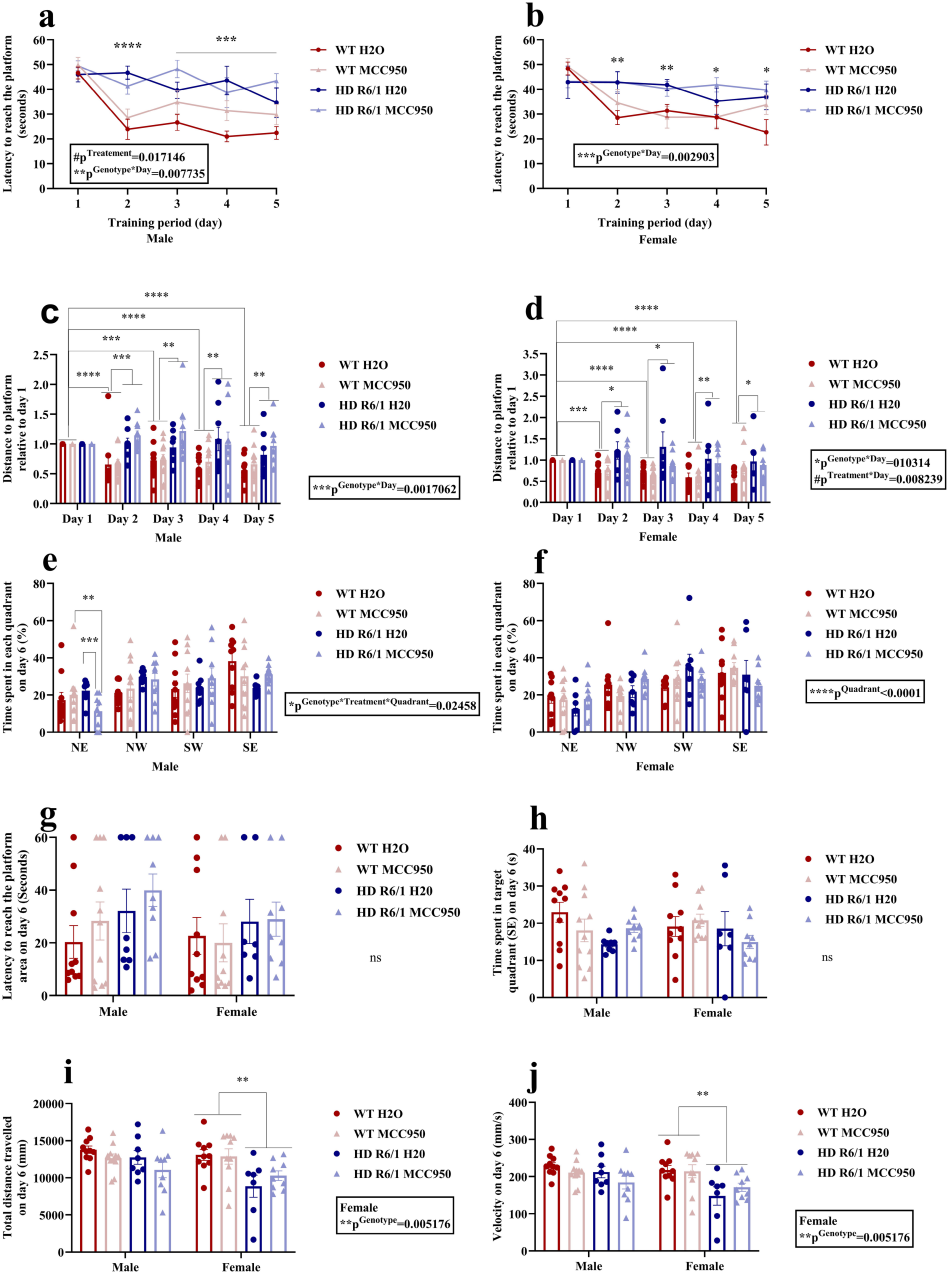
Effects of MCC950 on longer‐term associative cognition and spatial memory of HD and WT littermate control mice (MWM). Latency to reach the platform during training period of MWM test (a, b), distance travelled to reach the platform during training period of MWM test (c, d), time spent in each quadrant on day 6 of MWM test (e, f), latency to reach the platform on day 6 of MWM test (g), times spent in target quadrant on day 6 of MWM test (h), total distance travelled on day 6 of MWM test (i), velocity on day 6 of MWM test (j). Data are displayed as mean ± SEM. Statistical analyses were performed using linear mixed model repeated measure analysis/generalised LMM repeated measure analysis (a–f) and linear mixed model (LMM)/generalised LMM (g–j) followed by Bonferroni post hoc adjustment with emmeans package in R. *p* value (*α*) = 0.05 and ####,****p < 0.0001, ###,***p < 0.001, ##,**p < 0.01, #,*p < 0.05, ns= non‐significant. *n* = 8–11 (male), *n* = 7–10 (female). HD, Huntington's disease; MWM, Morris water maze; NE, northeast; NW, northwest; SE, southeast (target quadrant); SW, southwest; WT, wild type.

We also assessed the spatial memory and learning at 13 weeks of age by conducting the Morris water maze (MWM) test. Analysing the latency to find the hidden platform during the 5‐day training period, there was a significant interaction between genotype and training period (Figure 6a,b) both in males (*F*_(4,136)_ = 3.6217, *p* ^Genotype×Day^ = 0.007735) and females (*F*_(4,128)_ = 4.2509, *p* ^Genotype×Day^ = 0.002903). Post hoc analysis revealed that male HD control mice required more time to find the hidden platform from the 2nd to the 5th day during the training session of MWM. The same presentation was seen in female HD control mice compared to WT control mice. In males, we also observed a main effect of treatment (*F*_(1,34)_ = 0.017146, *p* ^Treatment^ = 0.007735) in latency of days training session (Figure 6a). We then assessed the distance travelled by the mice during the 5 days training (Figure 6c,d). Data were normalised relative day 1 performance and we found a significant interaction between genotype and day (Figure 6c,d) both in male (*χ* ^2^(4) = 17.2786, *p* ^Genotype×Day^ = 0.0017062) and female mice (*χ* ^2^(4) = 13.2055, ^*p*Genotype×Day^ = 0.010314). Post hoc analysis revealed that in both sexes, HD mice needed to swim longer distance in every day of training period to reach the hidden platform compared to the WT mice. In addition, compared to day 1, WT mice of both sexes a travelled shorter distance to reach the platform in the subsequent days (Figure 6c,d). In female mice, an interaction between treatment and day (*χ* ^2^(4) = 13.7215, *p* ^Treatment×Day^ = 0.008239) was also found but the post hoc test did not show any significant different between MCC950 treated and control mice. Then we analysed the data obtained from day 6 when the platform was removed from testing area target zone (SE). We compared the time spent in each quadrant from day 6 data, we found a significant interaction between genotype, treatment and quadrant (*F*_(3,136)_ = 3.2267, *p* ^Genotype×Treatment×Quadrant^ = 0.02458) in male (Figure 6e). Post hoc analysis revealed that there was no significant difference between groups in spending time in the targeted quadrant (SE), however, MCC950‐treated HD mice spent less time in NE (northeast) quadrant comparing to the control HD and MCC950‐treated WT mice. In females, a significant main effect of quadrant only (*F*_(3,128)_ = 10.6486, *p* ^Quadrant^ < 0.0001) was observed which did not indicate difference of genotype or treatment (Figure 6f). When we analysed latency to reach the hidden platform and time spent in the target quadrant from day 6 data, there was no significant difference observed in these parameters (Figure 6g,h). To further explore the locomotor impairment in water, total distance travelled, and velocity were analysed from day 6 data. We found a significant main effect of genotype for both distances travelled (*F*_(1,15.662)_ = 10.5390, *p* ^Genotype^ = 0.005176) and velocity (*F*_(1,15.662)_ = 10.5390, *p* ^Genotype^ = 0.005176) in females (Figure 6i,j) only, while male mice did not show any effect of treatment or genotype (Figure 6i,j). Post hoc analysis demonstrated that female HD mice travelled less distance their velocity was also decreased compared to WT mice during the day 6 (probe) trial in MWM test.

### 3.6 MCC950 Improved Faecal Output in Female HD Mice Only and Faecal Water Content and Faecal Consistency in Both Sexes of HD Mice

**FIGURE 7:**
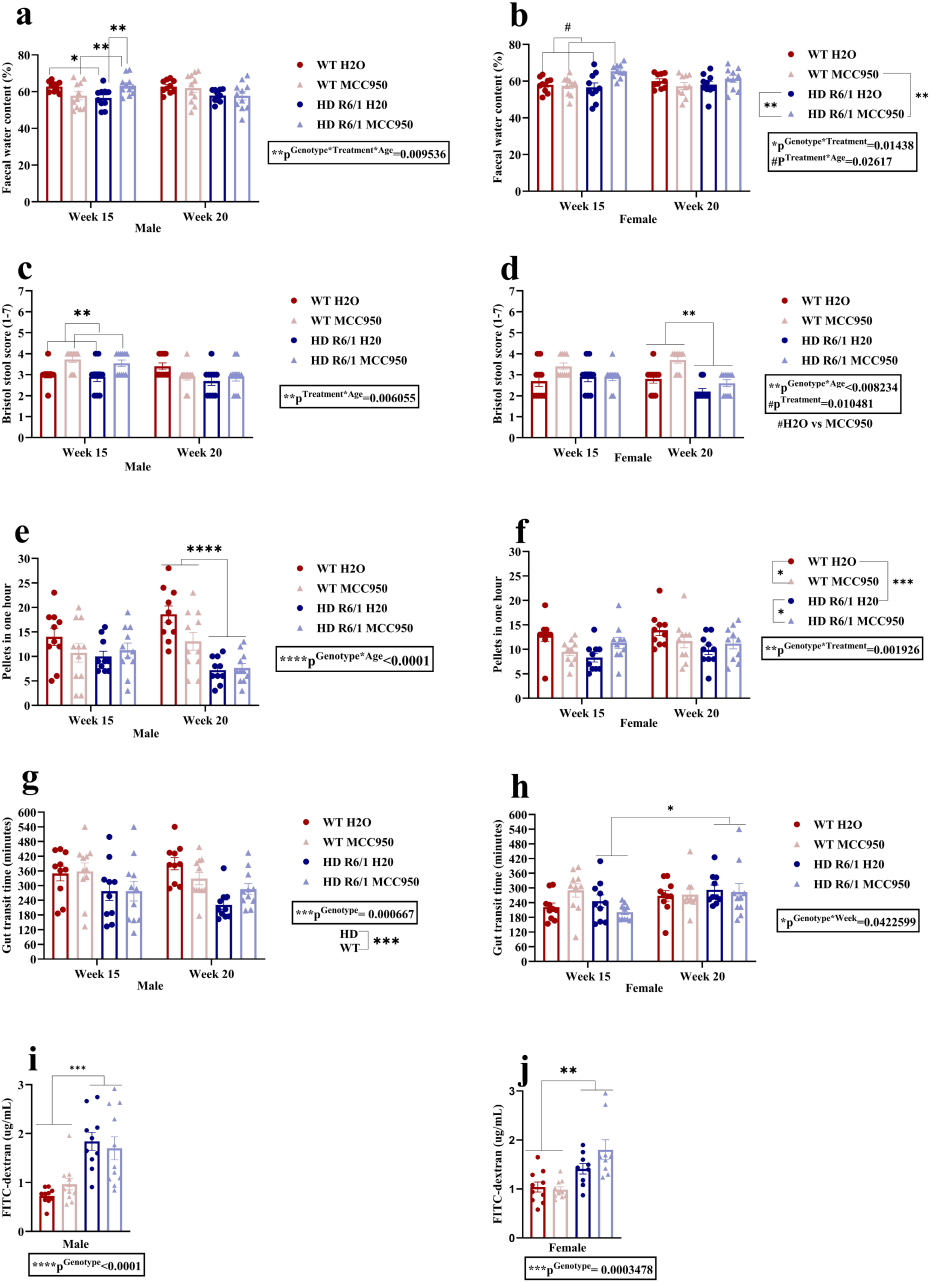
Effects of MCC950 on the gut profile of HD and WT littermate control mice. Faecal water content (a, b), faecal consistency (Bristol stool score) (c, d), faecal output (e, f), gut transit time (GTT) (g, h), gut permeability (i, j). Data are displayed as mean ± SEM. Statistical analyses were performed using linear mixed model repeated measure analysis/generalised LMM repeated measure analysis (a, b, e–h), CLMMs (cumulative link mixed models) (c, d), linear mixed model (LMM)/generalised LMM (i, j) followed by Bonferroni post hoc adjustment with emmeans package in R. *p*‐value (*α*) = 0.05 and ####,****p < 0.0001, ###,***p < 0.001, ##,**p < 0.01, #,*p < 0.05. *n* = 10–11 (male), *n* = 10 (female). HD, Huntington's disease; GTT, gut transit time; WT, wild type.

To evaluate gut function, we assessed the GTT, Bristol stool score, faecal water content and faecal output at 15 and 20 weeks of age. We observed a significant interaction of genotype, treatment and age (*F*_(1,38)_ = 7.4549, *p* ^Genotype×Treatment×Age^ = 0.009536) in faecal water content of males (Figure 7a). According to post hoc analysis, male HD control mice exhibited lower faecal water content compared to WT control mice at 15 weeks of age. However, MCC950‐treated HD mice showed an increase in faecal water content when compared to HD control and WT control groups (Figure 7a). At 20 weeks of age, post hoc analysis revealed that male HD mice did not significantly differ from their WT counterpart. With the faecal water content measurement in females, a significant interaction effect of genotype and treatment (*F*_(1,36)_ = 6.6154, *p* ^Genotype×Treatment^ = 0.01438) and treatment and age (*F*_(1,36)_ = 5.3787, *p* ^Treatment×Age^ = 0.02617) were observed (Figure 7b). Post hoc analysis of genotype and treatment interaction revealed that MCC950‐treated female HD mice displayed increased faecal water content compared to control HD and MCC950‐treated WT mice. Additionally, post hoc analysis of treatment and age interaction showed MCC950‐treated female mice at 15 weeks of age exhibited increased faecal water content than the control mice. Then we assessed the faecal consistency by evaluating Bristol stool scores ranging from 0 to 7. A significant interaction between treatment and age (*χ* ^2^(1) = 7.5338, *p* ^Treatment×Age^ = 0.006055) was observed in male mice (Figure 7c). Post hoc analysis demonstrated that at 15 weeks of age, male mice treated with MCC950 displayed increased stool consistency compared to the control mice. In females, a significant interaction between genotype and age (*χ* ^2^(1) = 6.9818, *p* ^Genotype×Age^ = 0.008234) was observed (Figure 7d). Post hoc analysis revealed that female HD mice at 20 weeks of age showed decreased Bristol stool scores compared to the WT mice. Female HD mice also showed decreased Bristol stool scores at 20 weeks of age when compared with 15 weeks of age. In addition, a main effect of treatment (*χ* ^2^(1) = 6.5513, *p* ^Treatment^ = 0.010481) was observed in female which indicated that MCC950‐treated mice showed increased Bristol stool scores compared to the non‐treated control groups (Figure 7d). The faecal output result exhibited an interaction between genotype and age (*F*_(1,38)_ = 26.9876, *p* ^Genotype×Age^ < 0.0001) in males (Figure 7e). Post hoc analysis showed HD mice had lower faecal output than the WT mice at 20 weeks of age. Compared to 15 weeks of age, the faecal output was decreased in HD mice but increased in WT mice at 20 weeks of age. In females, faecal output was significantly different between groups as we noticed an interaction effect of genotype and treatment (*F*_(1,120.05)_ = 10.3665 *p* ^Genotype×Treatment^ = 0.001926) (Figure 7f). Post hoc analysis revealed that female HD control mice had decreased faecal output than the WT control mice (Figure 7f). MCC950 significantly improved this faecal output in female HD mice when compared with control HD mice. In contrast, MCC950 had a different outcome in WT mice, where it decreased their faecal output compared to the WT control group (Figure 7f).

In GTT, a significant main effect of genotype was observed (*F*_(1,13.7398)_ = 38, *p* ^Genotype^ = 0.000667) in male mice, which indicated male HD mice in general had lower GTT than WT mice (Figure 7g). In females, we found a significant interaction between genotype and age (*χ* ^2^(1) = 4.1248, *p* ^Genotype×Age^ = 0.0422599) when we assessed the GTT (Figure 7h). Post hoc analysis showed that control HD mice had increased GTT at 20 weeks of age compared to week 15. MCC950 did not alter the GTT in any groups. We also noticed a significant increase in gut permeability in HD mice than WT mice in males (Figure 7i, *p* ^Genotype^ < 0.0001) and females (Figure 7j, *p* ^Genotype^ = 0.0003478). MCC950‐treated animals showed no difference in gut permeability regardless of genotype and sex.

### 3.7 MCC950 Modulated Colon Length in Female HD Mice and WT Mice

**FIGURE 8:**
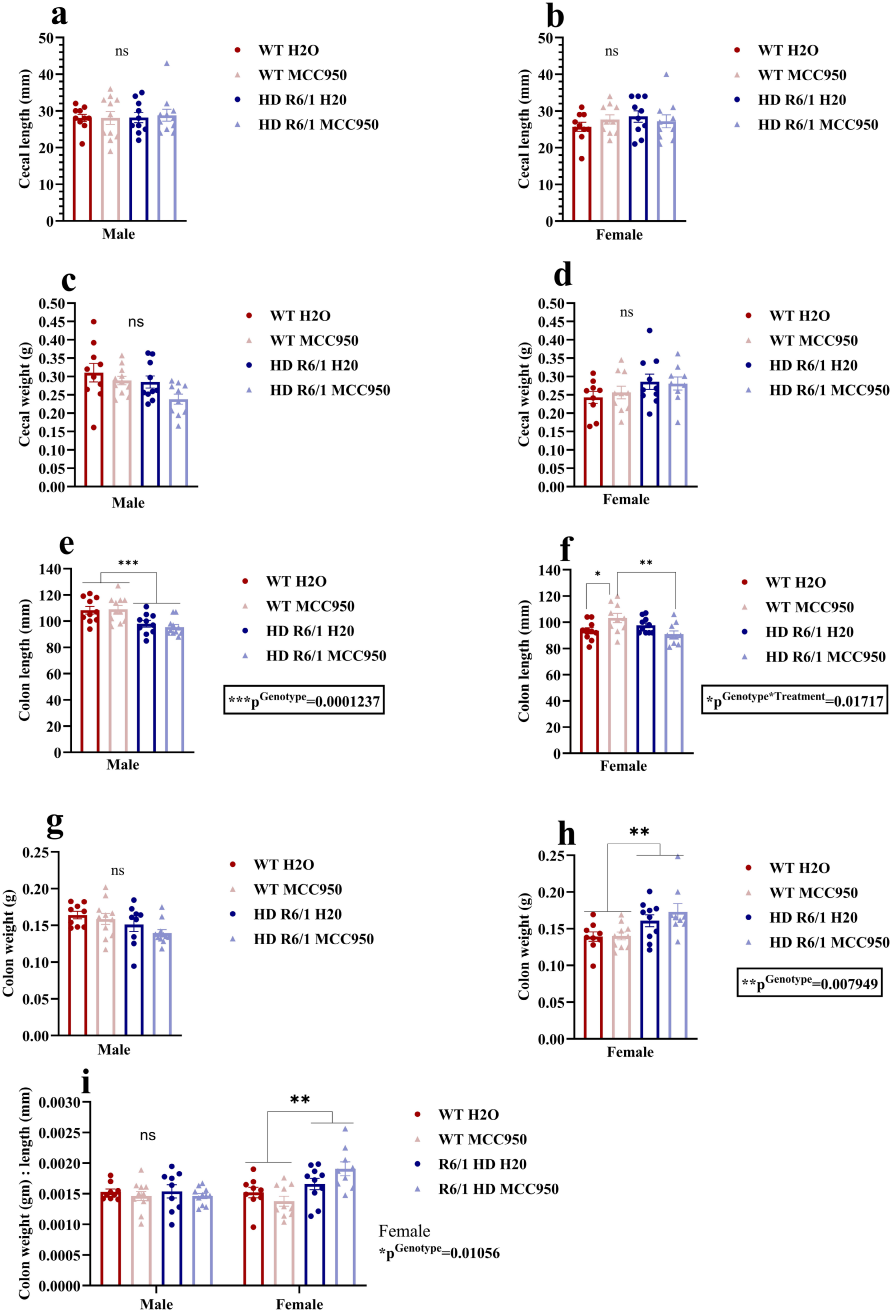
Effects of MCC950 on gut macroscopy of HD and WT littermate control mice. Cecal length (a, b), cecal weight (c, d), colon length (e, f), colon weight (g, h), colon weight to length ratio (i). Data are displayed as mean ± SEM. Statistical analyses were performed using linear mixed model (LMM)/generalised LMM followed by Bonferroni post hoc adjustment with emmeans package in R. *p* value (*α*) = 0.05 and ****p < 0.0001, ***p < 0.001, **p < 0.01, *p < 0.05, ns = non‐significant. *n* = 9–11 (male), *n* = 9–10 (female). HD, Huntington's disease; WT, wild type.

We evaluated the gut macroscopy parameters, including the length and weight of the cecum and colon. We did not find any difference in cecal length related to genotype or treatment (Figure 8a,b). The same applies to caecal weight in both sexes (Figure 8c,d). Regarding colon length, there was a main effect of genotype in males (*χ* ^2^(1) = 14.7357, *p* ^Genotype^ = 0.001237), suggesting HD male mice had shorter colon length compared to WT (Figure 8e). In females, a significant genotype and treatment interaction was found (*F*_(1,16)_ = 7.0667, *p* ^Genotype×Treatment^ = 0.01717) (Figure 8f). Post hoc tests suggested that the colon length was increased in MCC950‐treated WT mice compared to the control WT mice (Figure 8f). Interestingly, MCC950 increased the colon length in WT mice but not in HD mice. In males, a marginal effect of genotype (*F*_(1,15.804)_ = 4.0992, *p* ^Genotype^ = 0.06014) was observed for colon weight (Figure 8g). However, in females, a significant main effect of genotype (*χ* ^2^(1) = 7.0448, p^Genotype^ = 0.007949) was observed, indicating an increased weight of the colon in HD mice compared to WT (Figure 8h). Furthermore, colon weight to length ratio was evaluated as a proxy for gut inflammation (Figure 8i), and whilst in males there were no differences between groups, a significant main effect of genotype was found in females (*F*_(1,15.823)_ = 8.4029, *p* ^Genotype^ = 0.01056), suggesting that HD mice had more colon weight to length ratio (Figure 8i). MCC950 did not alter this measure.

### 3.8 MCC950 Did Not Improve HD Gut Microbiome Profile

**FIGURE 9:**
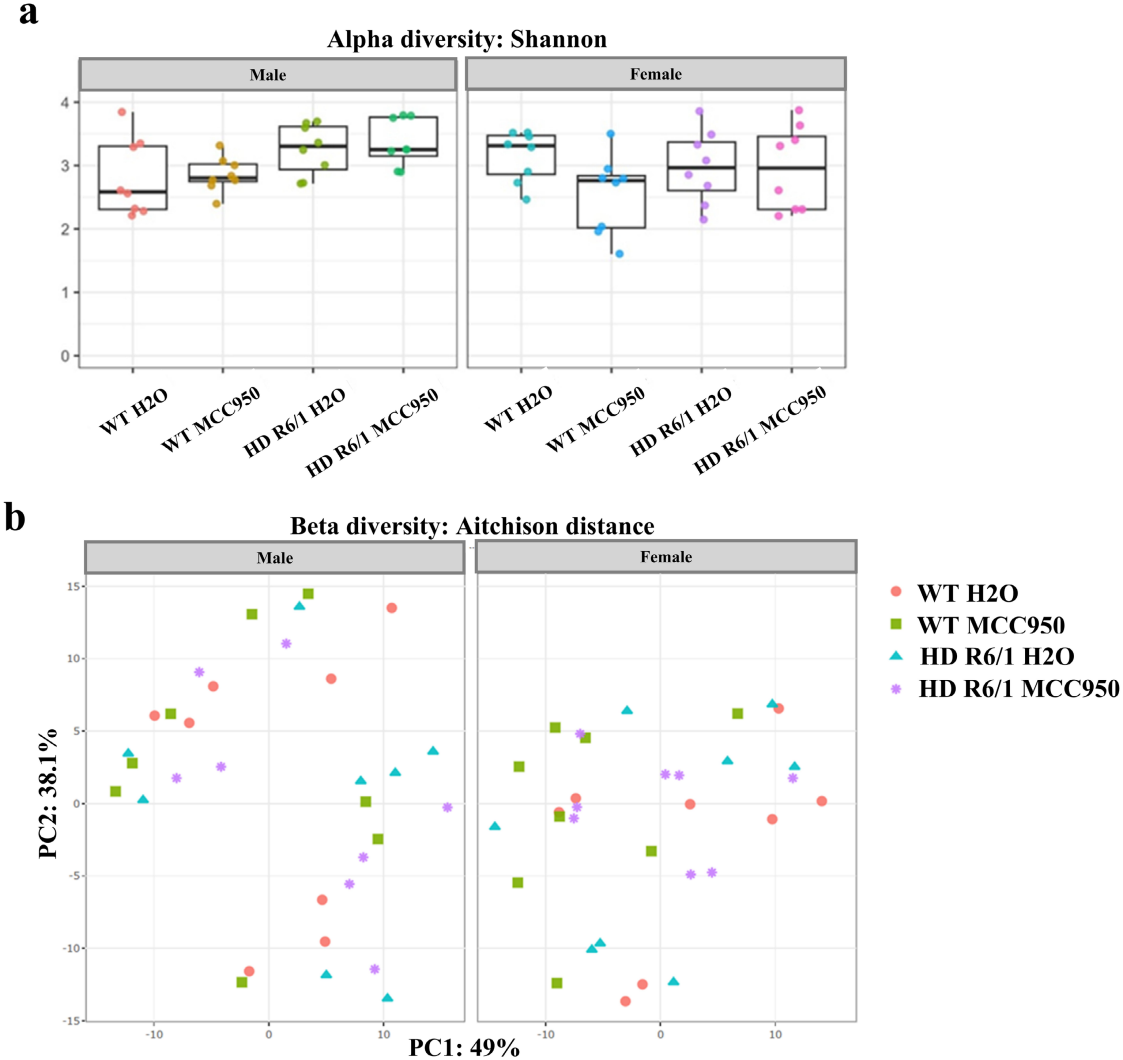
Effect of MCC950 on gut microbiome diversity of HD and WT littermate control mice. Comparison of community (Shannon alpha diversity) of gut microbiome stratified by sex under MCC950 or water for both WT and HD mice (a). PCA plot of microbial ASV based on Aitchison distance for Beta diversity (b). *n* = 8 (male), *n* = 8 (female). Box plots show the median (central line) and interquartile range (box); whiskers represent 1.5× the interquartile range; points represent individual animals. ASV, amplicon sequence variant; HD, Huntington's disease; PCA, principal component analysis; WT, wild type.

Comparison of microbial community (alpha diversity) of each group was stratified by sex. To assess the effect of genotype and treatment, a three‐way ANOVA was performed. There was a significant main effect of genotype on Shannon index (*F* = 5.3586950, *p* ^Genotype^ = 0.0243093), indicating that the microbial community in HD mice differed from WT mice (Figure 9a). Treatment with MCC950 did not modify this measure (F = 0.8546967, *p* ^Treatment^ = 0.35). To further evaluate within‐group differences, pairwise Wilcoxon tests (FDR‐corrected) were performed, but no significant pairwise comparisons were detectable. Next, to assess the beta diversity, the Aitchison distance was calculated and visualised (Figure 9b). PERMANOVA was conducted to evaluate the effects of genotype, treatment, and sex on microbiota variation. There was no effect of sex (*R* ^2^ = 0.0196107, *p* ^Sex^ = 0.298). Herein, HD mice did not significantly differ from WT mice (*R* ^2^ = 0.0308635, *p* ^Genotype^ = 0.131), nor did MCC950 alter beta diversity (*R* ^2^ = 0.0059024, *p* ^Treatment^ = 0.707).

### 3.9 MCC950 Downregulated Cleaved Caspase‐1 in Female HD Mice

**FIGURE 10:**
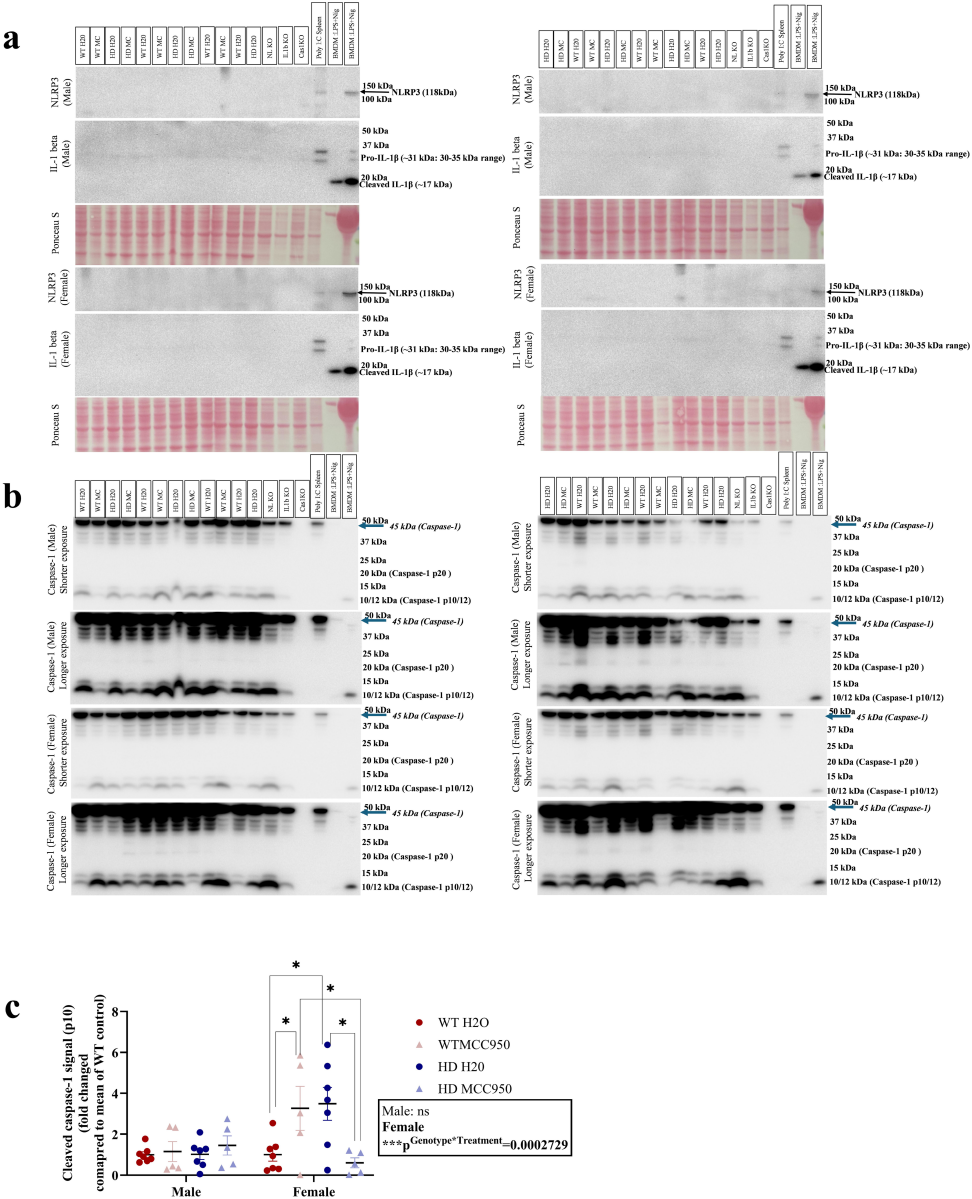
Effect of MCC950 on NLRP3, IL‐1β, Caspase‐1 expression in gut (proximal colon) of HD and WT littermate control mice. NLRP3 and IL‐1β (a), caspase‐1 (b, c). Data are displayed as mean ± SEM. Statistical analyses were performed using linear model (LM)/generalised LM followed by Bonferroni post hoc adjustment with emmeans package in R. *p* value (*α*) = 0.05 and ****p < 0.0001, ***p < 0.001, **p < 0.01, *p < 0.05, ns = non‐significant. *n* = 5–7 (2 blots for males and 2 blots for females). After transferring the protein from gel to membrane, ponceau S staining was performed and then each membrane was split into two parts based on the size of the protein of interest. Upper part of the membrane was probed with NLRP3 antibody. While lower part of the membrane was first probed with IL‐1β followed by caspase‐1. Membrane was stripped using stripping buffer in between two experiments of IL‐1β and caspase‐1 detection. Representative image of ponceau S staining as loading control for both NLRP3 and IL‐1β (a), and caspase‐1 (b) is shown in NLRP3 and IL‐1β (a) panel only as it was derived from a single membrane and image was captured before probing with any antibody. Positive controls, poly I:C (spleen), BMDM: LPS + Nig. BMDM, Bone‐marrow‐derived macrophage; Cas 1 KO, caspase‐1 knock out; HD H_2_O, Huntington's disease water; HD MC, Huntington's disease MCC950, NL KO, NLRP3 knock out; IL‐1beta KO, Interleukin 1 beta knock out; IL‐1β/IL‐1beta, Interleukin 1 beta; LPS, Lipopolysaccharides; Nig, nigericin; WT H_2_O, wild type water; WT MC, wild type MCC950.

Proximal colon samples were processed for western blot analysis to detect the expression of NLRP3, IL‐1β, and caspase‐1 (Figure 10a). Active expression of NLRP3 and IL‐1β was undetectable in the gut samples. Regarding the cleaved caspase‐1 expression (fold change), there was no difference between groups in male mice, and MCC950 did not affect any groups. However, a significant interaction effect of genotypes and treatments (*F*_(1,20)_ = 19.4060, *p* ^Genotype×Treatment^ = 0.0002729) was detected in females (Figure 10b,c). Post hoc analysis demonstrated that cleaved caspase‐1 was upregulated in HD control females compared to their WT counterpart. MCC950 decreased the expression of cleaved caspase‐1 in female HD mice compared to the HD control while it worked differently in female WT mice.

In addition, we also measured the ratio of cleaved caspase‐1 and procaspase‐1 in both males and females (Figure S3). In males, a significant main effect of genotype (*χ* ^2^(1) = 0.6190, *p* ^Genotype^ = 0.04424) was found which suggested that HD male mice had increased expression of cleaved caspase‐1 compared to the WT mice. MCC950 did not alter this parameter in males. In females, the genotype and treatment interaction effect (*F*_(1,20)_ = 7.454, *p* ^Genotype×Treatment^ = 0.0129) was significant and post hoc analysis revealed that MCC950 significantly downregulated the caspase‐1 in HD mice, but it acted differently in WT mice. Raw images of caspase‐1 expression in gut samples are provided in the Supporting Information (Figures S4–S6). Expression of NLRP3, IL‐1β, and caspase‐1 protein in non‐treated WT and HD control brain samples (hippocampus and striatum) was also evaluated. NLRP3, IL‐1β, and active caspase‐1 were below detectable range in HD mice at baseline by western blot analyses (Figure S7).

## 4 Discussion

Our findings indicate that NLRP3 inflammasome inhibition represents a promising strategy to ameliorate or delay gastrointestinal dysfunction in Huntington's disease. Continuous inhibition of NLRP3 inflammasomes with the oral administration of MCC950 modulated the gastrointestinal parameters. MCC950 administration increased the faecal water content in HD (in both sexes), correspondingly, it also softened the faecal consistency in both WT and HD subjects. These parameters are indicative of the water absorptive capacity of the gastrointestinal wall (Lewis and Heaton 1997), and our results reflected the alleviation of constipation and capacity for this approach to maintain healthy gut physiology. In addition to this, MCC950 increased the faecal output. Regarding the gut macroscopy, we also found that modulation of colon length due to the MCC950 treatment which occurred differently between WT and HD mice. Furthermore, molecular assessment of gut tissue by immunoblotting analyses demonstrated the downregulation of activated caspase‐1 in female HD mice which is downstream substrate of NLRP3 inflammasome pathway. Our findings have promising therapeutic value for modulating the peripheral symptoms of HD especially considering the debilitating gastrointestinal complications experienced by gene‐positive individuals. This is the first study to investigate gut health in HD by inhibiting NLRP3 inflammasome.

People living with HD often experience a series of gastrointestinal complications, including difficulty swallowing food, an inflamed erosive oesophagus frequently causing haemorrhages, difficult bowel movements, and constipation. These excruciating symptoms are commonly unaddressed and reduce the quality of life of people with HD (Kolachana et al. 2024). It is reported that gastrointestinal symptoms are often unreported by these individuals because of their inability to express the pain and suffering they experience due to other concurrent symptoms like dementia, leading to the worsening of the gastrointestinal symptoms (Andrich et al. 2009).

In this study, we characterised the HD phenotype in the R6/1 mouse model. R6/1 mice mimic adequate face and construct validity for HD with a progressive degenerative phenotype, as seen in human subjects. The phenotypic characteristics of R6/1 HD mice include loss of body weight, brain weight, and increased thirst. They also show impaired motor function (rotarod performance, clasping score, and gait assessment). The impaired short‐term and long‐term memory is also evident in HD mice. These features have been demonstrated in several previous studies (Gubert et al. 2021, 2022, 2023; Ekwudo et al. 2025). We corroborated these findings by showing that HD mice present with lower body weight. They also have motor abnormalities, increased water intake, and altered food consumption. In clasping score and rotarod performance, HD mice showed decreased motor activity compared to the WT mice. We also further confirmed that HD mice had an increased propel‐to‐brake ratio in the gait performance, which demonstrated their motor abnormality.

Gut function alterations are an emerging topic in HD and have been shown in several studies using R6/1 HD mice (Gubert et al. 2022, 2023). Additionally, this aspect of HD pathology (gastrointestinal complications) has been demonstrated widely in human subjects (Mehanna and Jankovic 2024). Evidence also suggests that gut dysbiosis is a crucial mediator of peripheral pathology in Huntington's disease (Wasser et al. 2020). The pioneering study led by Kong et al. (2018) demonstrated the occurrence of gut microbiota alterations in R6/1 HD mice before the onset of disease's salient features (Kong et al. 2018). This early alteration in gut microbiota may be pathogenic, possibly through plasma metabolite changes in HD (Kong et al. 2020). Moreover, the first‐ever clinical evaluation of gut dysbiosis in HDGECs (Huntington's Disease Gene Expansion Carriers) was by Wasser and colleagues in 2020, who discovered that gut microbiota alteration is related to poor clinical outcomes, particularly cognition in these subjects (Wasser et al. 2020). Targeting the gut microbiota in R6/1 HD mice as a therapeutic intervention exhibited significant positive outcomes after transplanting faecal microbiota from WT mice to HD mice (Gubert et al. 2022), administering a high‐fibre diet (Gubert et al. 2023) or a combination of prebiotics (Ekwudo et al. 2025). Interestingly, a study uncovered that altered plasma cytokine profiles are related to the changes in gut microbiota (Du et al. 2021). Furthermore, altered gut health and gut microbiota coexist in HD pathology (Gubert et al. 2022, 2023). For instance, along with the gut dysbiosis, and like the clinical symptomatology, HD mice have constipation and decreased gut motility and function as they exhibit decreased faecal water content (Ekwudo et al. 2025; Gubert et al. 2023) and faecal output (Gubert et al. 2022). Faecal consistency is also an important parameter to measure constipation via the Bristol stool score. During bowel movement, the amount of water reabsorption in the large intestine determines the faecal consistency (Lewis and Heaton 1997). In addition, HD mice demonstrate decreased gut transit time (Ekwudo et al. 2025; Gubert et al. 2023). They also have leaky gut as evidenced by the increased gut permeability (Ekwudo et al. 2025; Gubert et al. 2022, 2023). Intriguingly, another mouse model of HD (R6/2) with increased gut permeability was also shown to have altered gut microbiota (Stan et al. 2020). Regarding the NLRP3 inflammasome, many studies have suggested its involvement in brain pathology but rarely targeted its contribution to the peripheral pathology of neurodegenerative diseases associated with gut dysfunction and dysbiosis (Sarkar et al. 2025). Similarly, in HD, researchers found that NLRP3 inflammasome is upregulated in the PBMCs of clinical HD (Glinsky 2008) and in the brain samples of the R6/2 HD mouse model (Paldino, D'Angelo, Sancesario, and Fusco 2020). Therapeutic interventions targeting the NLRP3 inhibition have also shown improvement in the HD pathology (Paldino, D'Angelo, Laurenti, et al. 2020). However, no study to date has investigated the impact of NLRP3 inflammasome inhibition on gut health and gut microbiota alteration in HD.

Our study reproduced the alteration of gut microbiota in the R6/1 preclinical model of HD previously demonstrated (Ekwudo et al. 2025; Gubert et al. 2023). Herein, we found that HD mice differ from WT mice in terms of the alpha diversity (Shannon) of the microbial community. However, we did not detect a difference in the beta diversity of the gut microbiota in HD mice compared to WT mice. Our recent result partially aligned with the previous findings of gut microbiota alterations in HD, which showed both alpha and beta diversity of the intestinal microbial community significantly differ in HD mice compared to the WT (Ekwudo et al. 2025; Gubert et al. 2023; Kong et al. 2018). Here, we corroborated the existence of gut dysbiosis in HD; however, MCC950 treatment did not exhibit any effect on the alpha diversity (Shannon) or beta diversity of the gut microbiome in any group of mice compared to vehicle control groups.

The present study has shown that the compromised gastrointestinal measurements are evident in the R6/1 HD mouse model. We have reported that HD mice have lower gut transit time (in males), decreased faecal water content (in males), and output (in both sexes), along with harder stool (lower Bristol stool score). These altered parameters suggest impairment of gut functions in HD mice. We also report that HD mice have increased gut permeability, indicating intestinal epithelium disintegration. Moreover, we report the gut macroscopy measurements to further detect gastrointestinal complications in HD mice. We demonstrate that HD mice have decreased colon length (in both sexes) and increased colon weight (in females). Previous studies showed that HD mice have shorter colon length as compared to the WT control mice (Gubert et al. 2022, 2023). We also measured the colon weight to length ratio, which is an indirect measurement of gut inflammation (Carvajal et al. 2017; Kim et al. 2021). This current study demonstrates an increased colon weight to length ratio in female HD mice, which reinforces the concept of a disrupted gut health phenotype in HD mice.

We demonstrated increased expression of cleaved caspase‐1, a downstream effector of inflammasome activation, in the gut of HD mice compared with WT controls. Pharmacological inhibition of the NLRP3 inflammasome with MCC950 selectively reduced cleaved caspase‐1 levels in female HD mice, providing molecular evidence of inflammasome modulation in the gastrointestinal tract. Previous studies using R6/2 HD models have reported that inflammasome inhibition reduces caspase‐1 activation in the CNS (Chen et al. 2022; Paldino, D'Angelo, Laurenti, et al. 2020) and implicates caspase‐1 in HD‐related brain pathology (Chen et al. 2000; Ona et al. 1999). In contrast, we did not detect NLRP3 or IL‐1β protein expression in the hippocampus, striatum, or gut at the disease stage examined in R6/1 mice, suggesting model‐ or stage‐specific regulation of inflammasome components. Notably, to our knowledge, this is the first study to implicate caspase‐1 activation in gut pathology in a Huntington's disease model. The sex‐specific efficacy of MCC950, which reduced caspase‐1 activation in females but not males, highlights sexual dimorphism in inflammasome‐related signalling in HD. These findings suggest that NLRP3‐dependent caspase‐1 activation contributes to peripheral inflammatory pathology in HD in a sex‐dependent manner, while alternative inflammatory pathways may predominate in males. Together, our data indicate that inflammasome inhibition effectively modulates peripheral pathology but may be insufficient to reverse central disease phenotypes in this model.

Given the marked improvement in gastrointestinal function following MCC950 treatment, we next investigated whether inflammasome‐related molecular pathways contribute to gut pathology in HD. In our study, MCC950 exhibited the capacity to significantly enhance the faecal water content (in both males and females) and faecal output (in females), as well as faecal consistency (in both sexes) of HD mice. Our recent findings are important considering the gastrointestinal complications that people living with HD endure. We have identified a novel therapeutic approach to recover gut health in HD, which may improve the overall well‐being of HD patients. To the best of our knowledge, no study has yet inhibited the NLRP3 inflammasome as a strategy to modulate altered gastrointestinal functions and pathology that arise from HD.

We assessed anhedonia, a key indicator of depression‐like behaviour, by measuring saccharin preference. In this experiment, saccharin preference of HD mice did not differ from WT mice. This finding is consistent with the previous study by Mo, Renoir, and Hannan (2014) who reported that HD mice did not display a difference in saccharin preference compared to WT mice. Increased thirst in HD mice could explain not having a difference in this test, which also makes the interpretation challenging (Mo, Renoir, and Hannan 2014). Besides, like human patients (Mitchell et al. 2005), changes in the taste perception may also contribute to the outcome of the saccharin‐preference test in HD mice, although it has not yet been explored (Mo, Renoir, and Hannan 2014). Future experiments may provide a better understanding using an alternative depressive‐like test. However, MCC950 did not modulate the performance of any group in this anhedonia‐like behaviour test. We also evaluated anxiety‐like behaviour in HD mice via the novelty‐suppressed feeding test. We found that HD males lost less body weight than WT, from the fast preceding the behaviour test, which might confound the primary anxiety‐related parameter of latency to feed. In females, the similar body weight loss prerequisite was met. HD mice did not show any symptomatology of anxiety in this test when compared to wild‐type mice in either sex. Previous findings showed HD female mice took longer to initiate feeding during this test (Renoir et al. 2011), which is an indicator of the rodent anxiety‐like behaviour hyponeophagia. Neither HD nor WT mice were influenced by MCC950 treatment in this anxiety‐like behavioural test performance.

To evaluate the short‐term cognitive changes in HD mice, we conducted the Y‐maze test. There was no difference between WT and HD mice when we analysed the total distance travelled, which means the measurement of the cognitive component would not be affected by locomotion and movement. HD mice did not show any alterations in novel arm preference compared to WT mice. However, we found a genotype effect in males that indicated HD mice left the home arm sooner than WT mice. In females, a trending genotype effect indicated that HD females took longer to leave the home arm, potentially an anxiety‐related result. While a significant treatment effect showed that MCC950‐treated mice were quicker to leave the home arm towards the central arena, indicating MCC950 was able to prevent the occurrence of this anxiety‐like behaviour in female HD mice. Like the male HD genotype effect seen here, a previous study observed that HD mice were quicker to leave the home arm of the Y‐maze test (Mo, Pang, et al. 2014). In addition, we conducted the novel‐object recognition test, which is a hippocampal‐independent cognitive task. In this test, there was no effect of genotype on object recognition or discrimination index, meaning HD mice did not show any differences in this type of memory test compared to WT mice. Our findings from this test align with a previous study that reported no significant change in hippocampal‐independent cognitive function in HD mice (Nithianantharajah et al. 2008).

Inhibition of NLRP3 inflammasome has been shown to improve motor function evaluated by clasping score and rotarod performance in R6/2 HD mice; it also improved body weight (Chang et al. 2025; Paldino, D'Angelo, Laurenti, et al. 2020). However, in our study, with the R6/1 model, NLRP3 inhibition by MCC950 did not alter these parameters. Differences in the animal model might be a cause of the non‐reproducibility of these findings. The R6/2 HD mouse model is faster progressing, with a higher number of CAG repeats expansion (around 150–210), displays rapid onset of HD symptoms and better models the juvenile form of HD (approximately 5% of patients). On the other hand, our model (R6/1) reflects adult onset of disease and slow progression of HD pathology and is ideal for studying the adult form of HD (approximately 95% of patients), as well as chronic interventions (Li et al. 2005; Quarrell et al. 2012; Roos 2010). Most importantly, no study has previously investigated the NLRP3 inflammasome in the R6/1 model; all previous studies investigated this pathological feature in R6/2 HD mice only. Furthermore, these studies used different drugs other than MCC950 to block the NLRP3 inflammasome in R6/2 HD mice (Chang et al. 2025; Paldino, D'Angelo, Laurenti, et al. 2020). However, MCC950 has been extensively used outside of the HD field to inhibit the NLRP3 inflammasome, and several studies have shown that it can effectively inhibit NLRP3 in mouse models of different diseases. Mice that received MCC950 versus vehicle (water) showed no adverse effect in their palatability (Gordon et al. 2018; Ioannou et al. 2023; Khan et al. 2017; Zhang et al. 2018). MCC950 by drinking water at a dose of 0.3 mg/mL, has similar effects in the blood (Gordon et al. 2018) as by other routes, including orally by gavage (Khan et al. 2017) and intraperitoneal administration (Coll et al. 2015; Marín‐Aguilar et al. 2020; Primiano et al. 2016; Qu et al. 2019). Treatment for 5 days of MCC950 at 0.3 mg/mL in drinking water showed good brain (29.3 ng/g) and plasma (5.86 μg/mL) concentration levels (Gordon et al. 2018). We followed the dose paradigm used by Gordon et al. 2018 and they reported that inhibition of NLRP3 inflammasome by oral administration of MCC950 effectively alleviated motor deficits, dopaminergic neurodegeneration and accumulation of α‐synuclein in PD mouse brain (Gordon et al. 2018). However, these researchers (Gordon et al. 2018) did not investigate all aspects of behavioural tests including depression and anxiety‐like behaviour, cognitive functions, which could be a possible explanation for challenges in reproducing and validating the efficacy of NLRP3 inflammasome inhibition.

Here, we also demonstrated the long‐term memory impairment in HD mice that includes poor performance in the fear conditioning and extinction test, and in the MWM test (latency to target platform). As an indicator of gross neurodegeneration in HD mice, we have demonstrated a decrease in whole brain weight of R6/1 HD mice. MWM cognitive performance was impaired in HD mice as shown in the R6/1 HD transgenic model with lower brain mass than their non‐transgenic counterpart (Lim et al. 2014). Impairment of memory and learning in HD mice was demonstrated in the fear conditioning and extinction test previously (Gubert et al. 2023; Ekwudo et al. 2025). Nevertheless, the effects of NLRP3 inflammasome inhibition on cognitive functions have not been evaluated earlier in HD. However, in other neurodegenerative disease studies, it showed a promising outcome of restoring impaired cognition in rodent models. For instance, NLRP3 inflammasome inhibition by MCC950 (50 mg/body weight) improved cognitive performance in a rodent model of Alzheimer's disease (AD) (Naeem et al. 2024). Additionally, MCC950 (10 mg/kg body weight, administered intraperitoneally) also improved cognition in a mouse model of MS, as evidenced by enhanced performance in the MWM and fear conditioning tests (Hou et al. 2020). Our study also demonstrated that HD mice performed poorly in the MWM test, as evidenced by the increased latency to reach the target platform. Furthermore, HD mice travelled a longer distance during the training days to reach the hidden platform compared to the WT mice. In addition, for the acquisition of memory learning from the training days, WT mice travelled less in the subsequent days to explore the hidden platform compared to the baseline, while HD mice did not. Moreover, HD mice did not show any differences in the latency to reach the platform and time spent in the target quadrant during day 6 (probe day). We further assessed the locomotory activity in the MWM to evaluate if any changes in motor functions were responsible for not showing the difference between WT and HD mice in the MWM cognitive task on Day 6. We found that HD mice had decreased total distance travelled parameter along with slower velocity compared to WT mice, which indicated that HD mice have motor abnormalities that potentially influence the cognitive result of the MWM test in terms of both genotype and MCC950 treatment effect. In the fear‐extinction test, HD mice show a partial failure to retain the associated memory (they did not freeze close to the level they did by the end of the conditioning), and then they did not appear to learn extinction, as they froze to a similar degree throughout the experiment. However, MCC950 did not improve these parameters across the groups in either sex. Although route‐specific effects could theoretically influence the efficacy of MCC950 on central phenotypes, oral administration via drinking water has been shown to achieve brain and plasma concentrations comparable to other routes, including intraperitoneal delivery (Gordon et al. 2018). Moreover, MCC950 administered orally has demonstrated central efficacy in other neurodegenerative models. These findings suggest that limited brain exposure alone is unlikely to explain the lack of cognitive and motor improvement observed here. Instead, our results indicate that selective NLRP3 inhibition may be insufficient to ameliorate complex CNS pathology in Huntington's disease, potentially due to compensatory inflammatory or inflammasome‐independent mechanisms.

## 5 Conclusion

In summary, we reproduced the gastrointestinal and microbial alterations previously reported in Huntington's disease mice. HD animals showed reduced microbial α‐diversity and multiple indicators of gut dysfunction, including slower transit, decreased faecal water content and output, and harder stools. Structural changes such as shortened colon length and increased colon weight, particularly in females, further supported the presence of intestinal pathology. Treatment with the NLRP3 inflammasome inhibitor MCC950 effectively improved several of these parameters, enhancing faecal consistency, water content, and output without altering overall microbiota diversity. These findings identify NLRP3 inhibition as a novel therapeutic approach to ameliorate gastrointestinal complications in HD, with potential to improve patients' gut health and overall wellbeing.

## Author Contributions

**Sujan Kumar Sarkar:** writing – original draft, writing – review and editing, formal analysis, methodology, investigation, validation, data curation, visualisation, conceptualisation. **Millicent N. Ekwudo:** investigation, writing – review and editing. **Da Lu:** investigation, writing – review and editing. **Bethany Masson:** investigation, writing – review and editing. **Pamudika Kiridena:** investigation, writing – review and editing. **Nicholas van de Garde:** investigation, writing – review and editing. **Thibault Renoir:** breeding and generation of experimental animals, writing – review and editing. **James E. Vince:** methodology, data curation, resources, writing – review and editing, validation. **Veerasikku Gopal Deepagan:** investigation, methodology, validation. **Anthony J. Hannan:** conceptualisation, funding acquisition, writing – review and editing, project administration, supervision, resources, data curation, software. **Carolina Gubert:** conceptualisation, funding acquisition, writing – review and editing, methodology, project administration, resources, supervision, data curation, software.

## Funding

C.G. is supported by a Bethlehem Griffiths Research Foundation (BGRF) grant. A.J.H. is supported by National Health and Medical Research (NHMRC) Ideas Grant funding, an EU‐JPND Grant, an ERA‐NET NEURON Grant, the Flicker of Hope Foundation, the DHB Foundation (Equity Trustees), and the Margaret Friend Trust. S.K.S. is supported by the Melbourne Research Scholarship (fee offset for PhD study) and Studentship (stipend) from the University of Melbourne, Australia, and the Science and Technology Fellowship Trust, Ministry of Science and Technology, Government of the People's Republic of Bangladesh. T.R. currently holds a Ronald Philip Griffiths Fellowship from the University of Melbourne.

## Conflicts of Interest

The authors declare no conflicts of interest.

## Supporting information


**Appendix S1:** jnc70419‐sup‐0001‐FigureS1‐S7.pdf.

## Data Availability

Data will be available on request.
