# Supplementary material for: Inhibition of the NLRP3 Inflammasome With MCC950 Improves Gut Health in Huntington's Disease Mice
Source: J Neurochem. 2026 Mar 30;170(4):e70419. doi: 10.1111/jnc.70419 (PMC13033966; doi:10.1111/jnc.70419)
Supplement: Supplementary file 1 — Appendix S1: jnc70419‐sup‐0001‐FigureS1‐S7.pdf. [file JNC-170-e70419-s001.pdf]

Inhibition of the NLRP3 inflammasome with MCC950 improves gut health in Huntington's disease mice

Sujan Kumar Sarkar[1,2,3], Millicent N. Ekwudo[1,2], Da Lu[1,2], Bethany Masson[1,2], Pamudika Kiridena[1,2], Nicholas van de Garde[1,2], Thibault Renoir[1,2], James E. Vince[4,5], Veerasikku Gopal Deepagan[4,5], Anthony J. Hannan[1,2,#], Carolina Gubert[1,2, #]

1. *Florey Institute of Neuroscience and Mental Health, Parkville, Australia.*
2. *Florey Department of Neuroscience and Mental Health, University of Melbourne, Parkville, Australia.*
3. *Department of Anatomy, Histology and Physiology, Faculty of Animal Science and Veterinary Medicine, Sher-e-Bangla Agricultural University, Dhaka, Bangladesh.*
4. *The Walter and Eliza Hall Institute of Medical Research, Parkville, VIC, Australia*
5. *Department of Medical Biology, University of Melbourne, Parkville, VIC, Australia*

Correspondence: Prof. A.J. Hannan or Dr C. Gubert,

Florey Institute of Neuroscience and Mental Health, University of Melbourne, Melbourne Brain Centre, Parkville VIC 3010, Australia

#These authors contributed equally to this work

E-mail: anthony.hannan@florey.edu.au, carolina.gubert@florey.edu.au

Supplementary materials

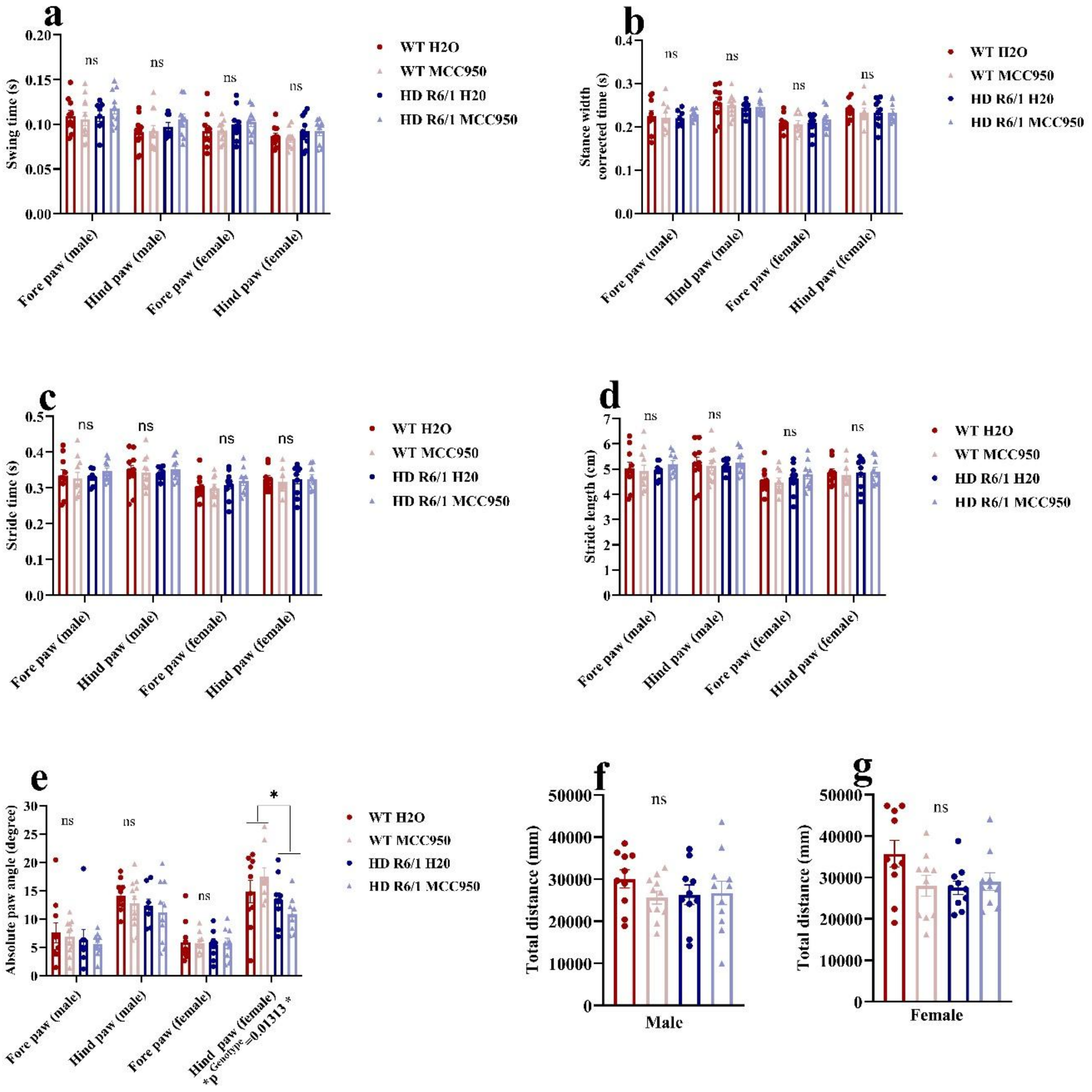

Supplementary figure 1 Effect of MCC950 on digigait performances (a-e), total distance travelled in Y-maze trial 1 (f-g) of HD and WT littermate control mice. Data were visualized using GraphPad prism-version 10.4.2 (633) and displayed as Mean+/-SEM, n=8-10 (male), n=9-10 (female) for digigait, n=10-11 (male), n=10 (female) for Y-maze. Statistical analyses were performed using linear mixed model (LMM)/Generalized LMM followed by Bonferroni post hoc adjustment with emmeans package in R (version 4.3.3) and R studio (version 2025.05.1+513). p value (α)=0.05 and ****p<0.0001, ***p<0.001, **p<0.01, *p<0.05. mice n=10-11 (male), n=10 (female). Abbreviations: WT, wild type; HD, Huntington's disease.

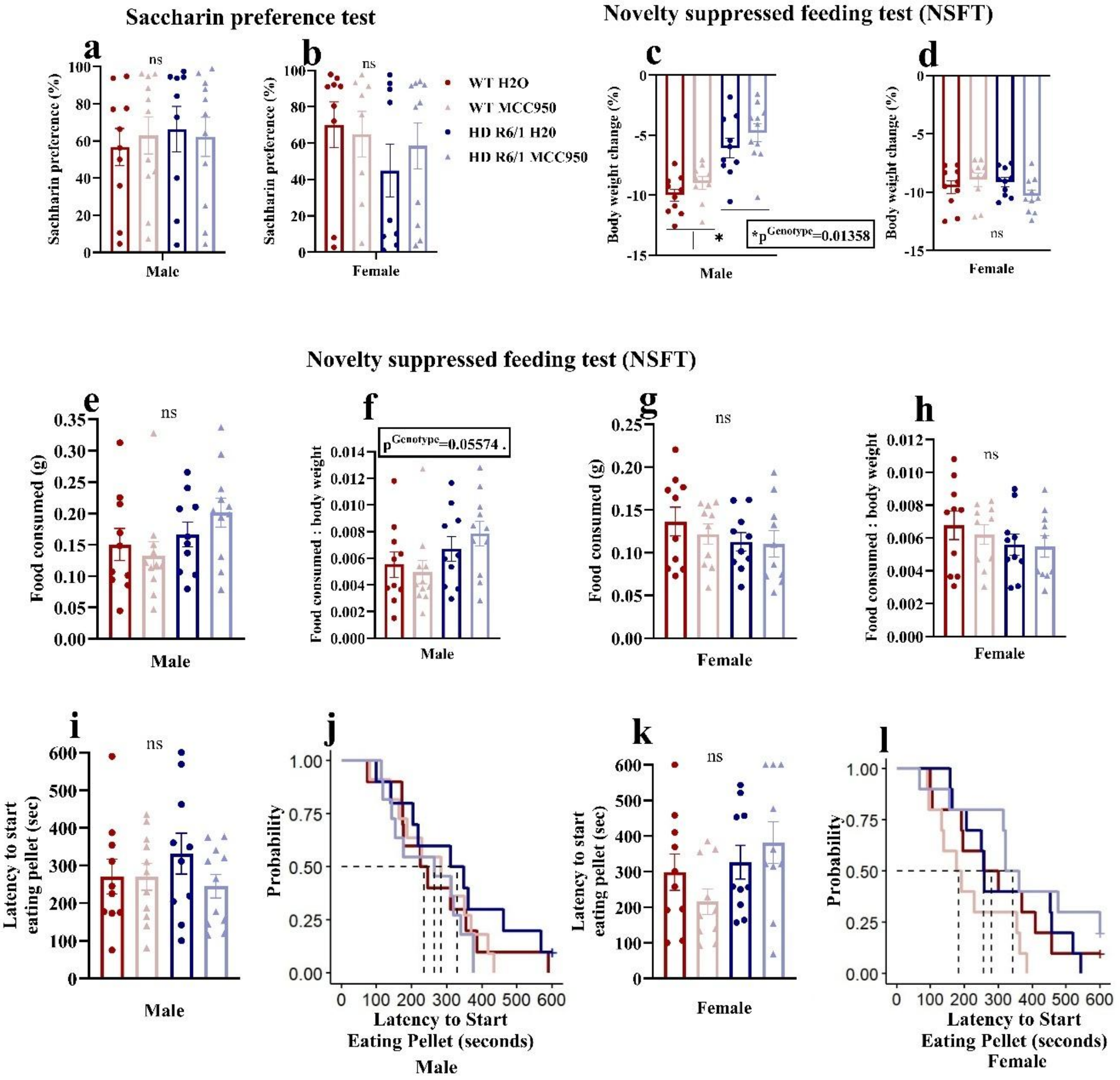

Supplementary figure 2 Effects of MCC950 on depression-like and anxiety-like behaviour of HD and WT littermate control mice. Saccharin preference test (SPT) (a-b), Novelty-suppressed feeding test (NSFT) (c-l). Data are displayed as mean ± SEM, n=9-11 (male), n=8-10 (female) for SPT, n=10-11 (male), n=10 (female) for Y-maze for NSFT. Statistical analyses were performed using linear mixed model (LMM)/generalized LMM followed by Bonferroni post hoc adjustment with emmeans package in R. For NSFT latency (j, l) Kaplan-Meier survival curves analysis was also performed. p value (α)=0.05 and ****p<0.0001, ***p<0.001, **p<0.01, *p<0.05. n=10-11 (male), n=10 (female). Abbreviations: WT, wild type; HD, Huntington's disease.

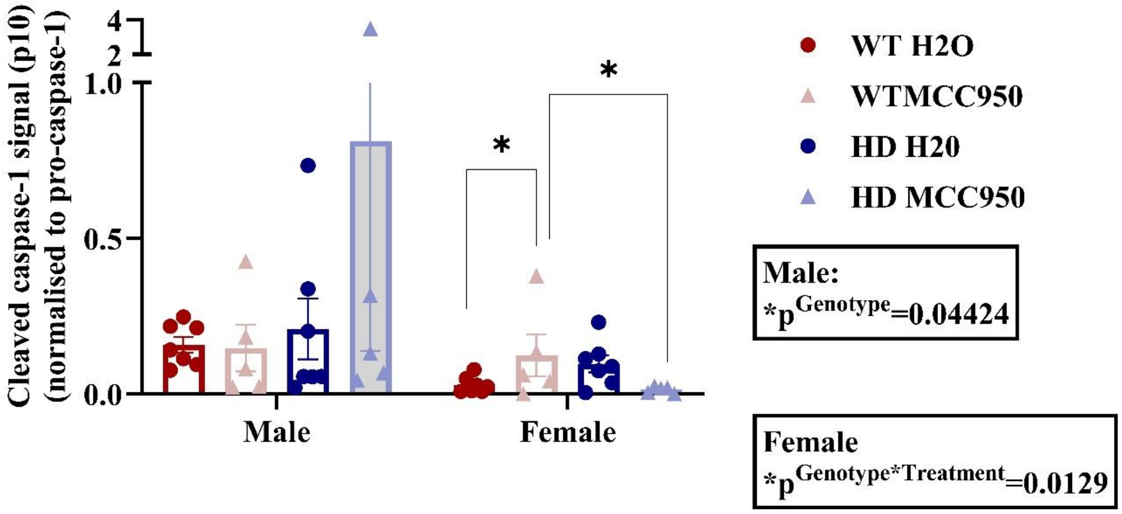

Supplementary figure 3 Effect of MCC950 on the ratio of cleaved caspase-1 and procapse-1 in both males and females. Data are displayed as mean ± SEM. Statistical analyses were performed using linear model (LM)/generalized LM followed by Bonferroni post-hoc adjustment with emmeans package in R. p value (α)=0.05 and ****p<0.0001, ***p<0.001, **p<0.01, *p<0.05. n=5-7 (2 blots for males and 2 blots for females). Abbreviations: WT, wild type water; HD, Huntington's disease. In male, a significant main effect of genotype (χ^2 (1) =0.6190, $p^{Genotype}$=0.04424) was found which suggested that HD male mice had increased expression of cleaved capsase-1 comparing to the WT mice. MCC950 did not alter this parameter in males. In females, genotype and treatment interaction effect (F (1, 20) = 7.454, $p^{Genotype*Treatment}$=0.0129) was significant and post hoc analysis revealed that MCC950 significantly downregulated the caspase-1 in HD mice, but it acted differently in WT mice.

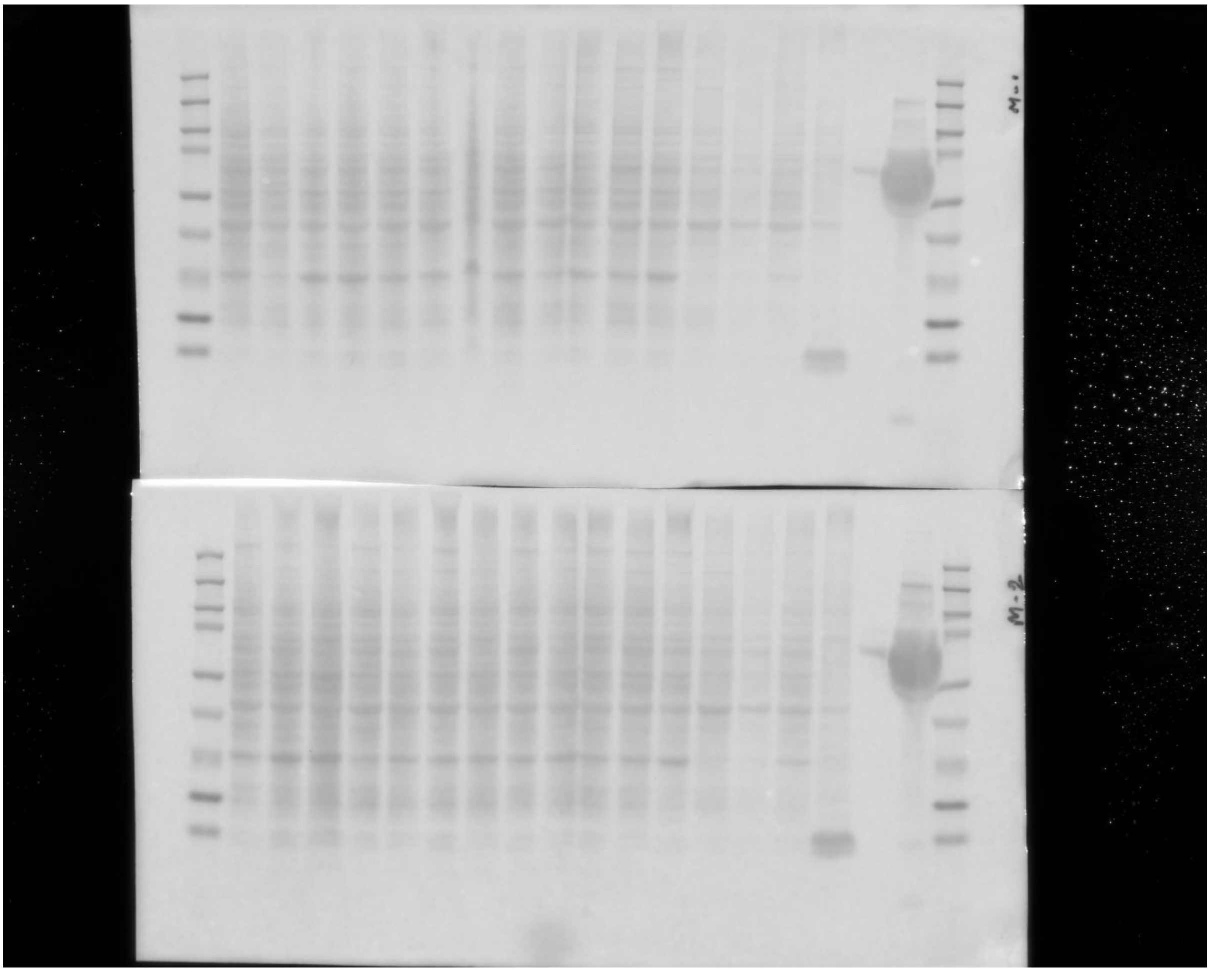

Supplementary figure 4 Total protein colorimetric picture (raw) after ponceau stain before probing with any antibody. Male (blot 1 up; blot 2 down), corresponds to the serial of samples in main **Figure 10** (2 blots side by side).

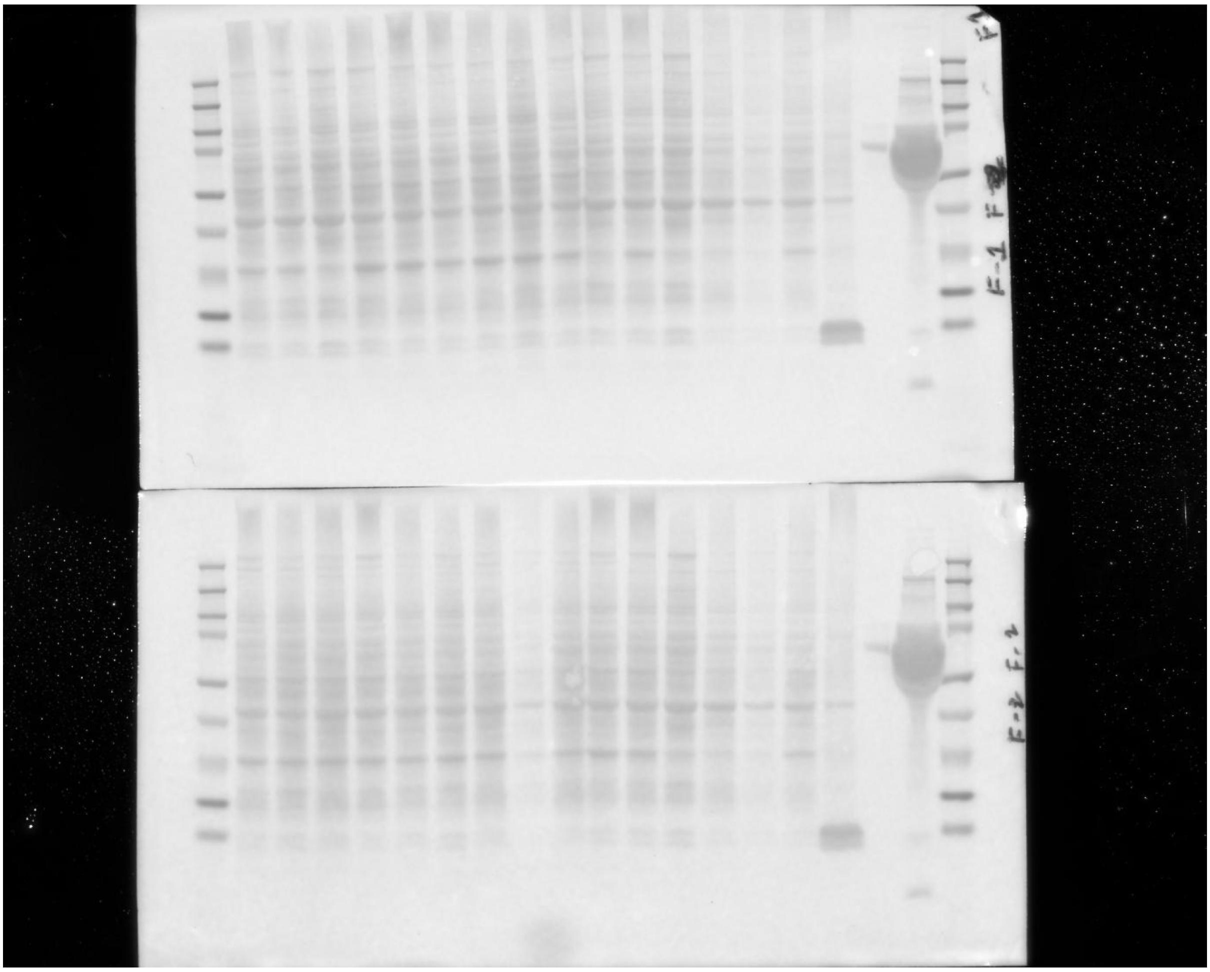

Supplementary figure 5 Total protein colorimetric picture after ponceau stain before probing with any antibody. Female (blot 1 up; blot 2 down), corresponds to the serial of samples in main **Figure 10** (2 blots side by side).

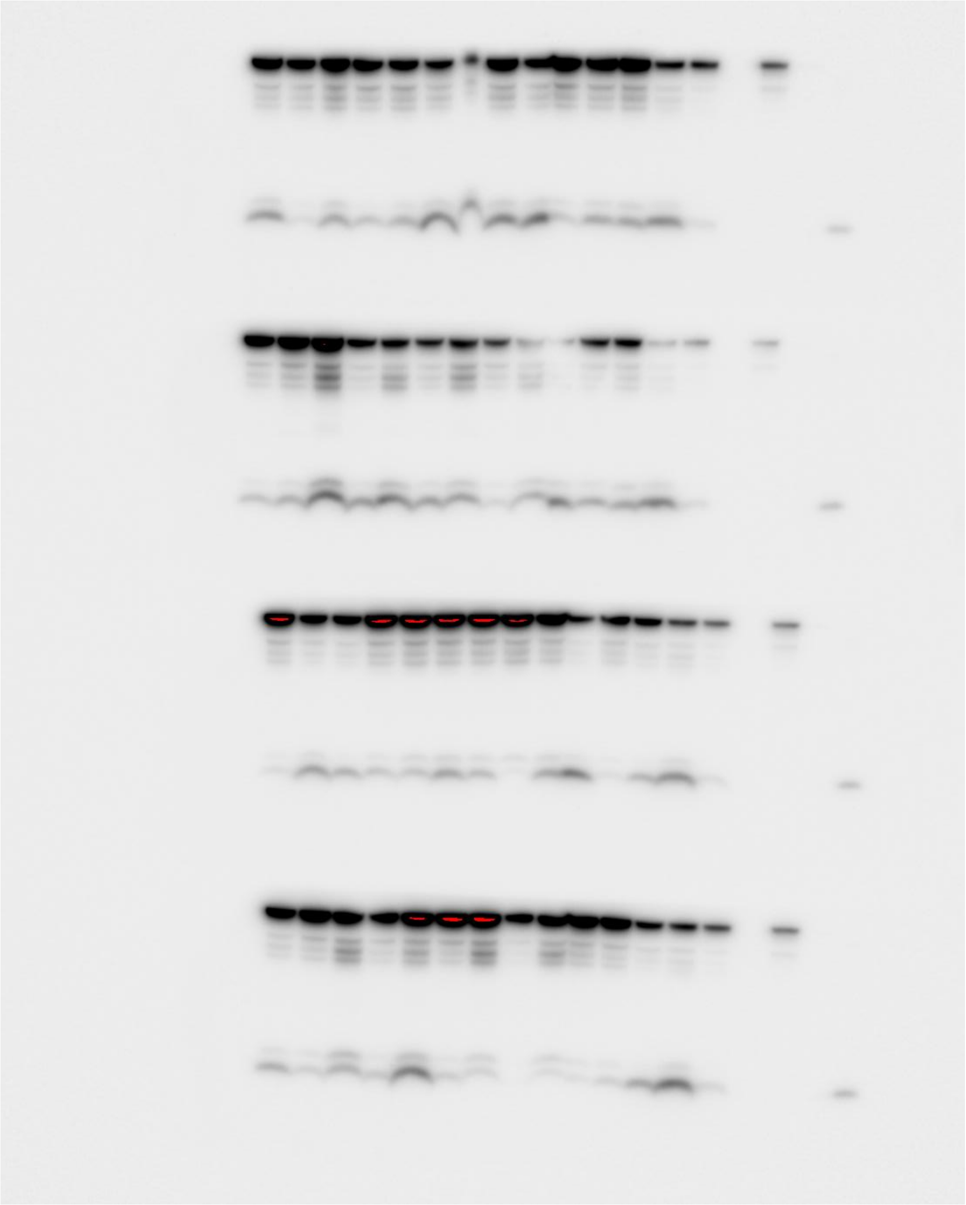

Supplementary figure 6 Raw chemiluminescence pictures of caspase-1 expression in gut samples. Four blots (first two males and last two females) were captured together. Blots correspond to the serial of samples in main **Figure 10** (2 blots side by side for each sex).

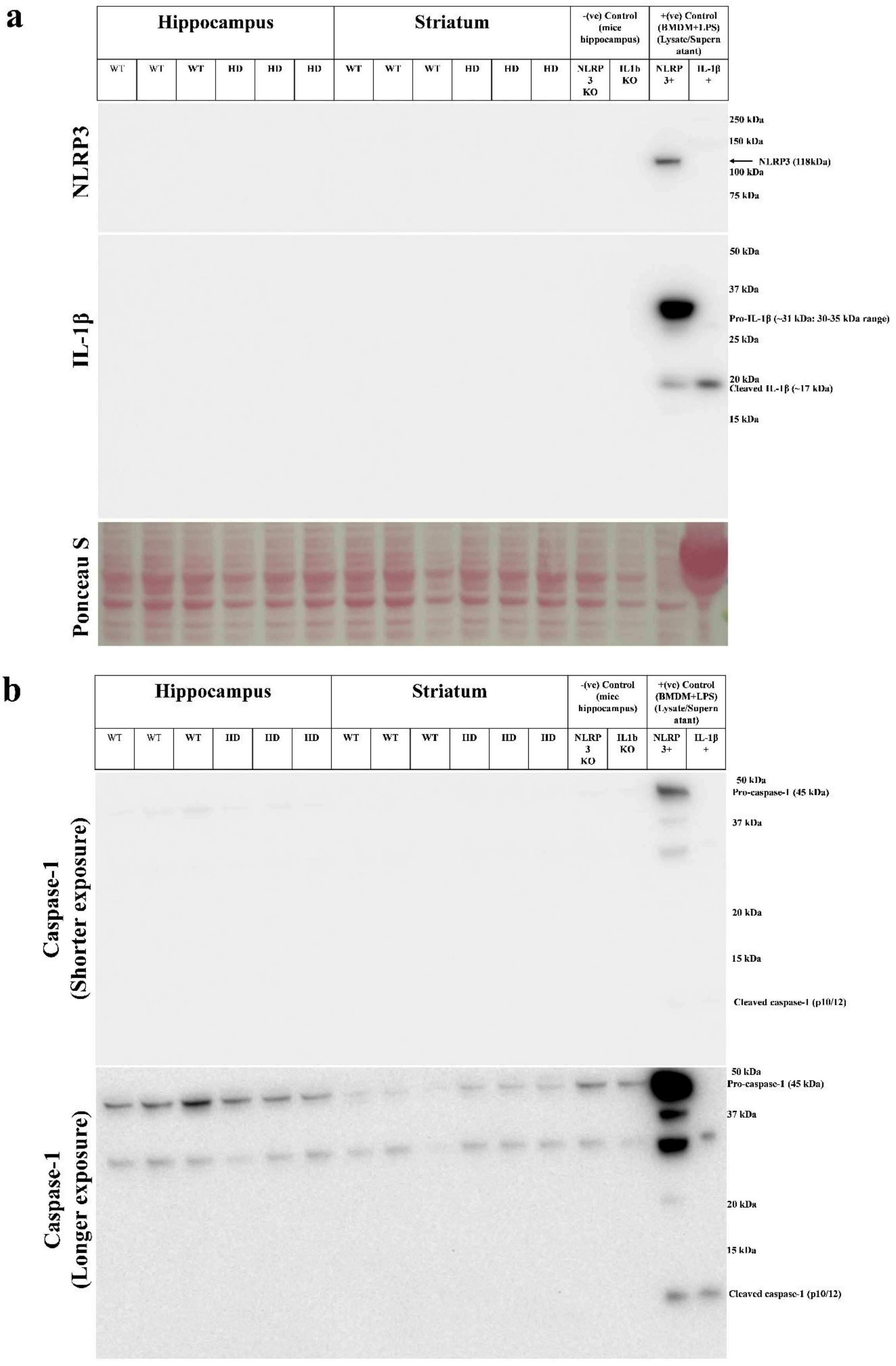
a
Hippocampus
Striatum
-(ve) Control (mice hippocampus)
+(ve) Control (BMDM+LPS) (Lysate/Supernatant)
WT
WT
WT
HD
HD
HD
WT
WT
WT
HD
HD
HD
NLRP 3 KO
IL1b KO
NLRP 3+
IL-1β +
NLRP3
250 kDa
150 kDa
NLRP3 (118kDa)
100 kDa
75 kDa
IL-1β
50 kDa
37 kDa
Pro-IL-1β (~31 kDa: 30-35 kDa range)
25 kDa
20 kDa
Cleaved IL-1β (~17 kDa)
15 kDa
Ponceau S
b
Hippocampus
Striatum
-(ve) Control (mice hippocampus)
+(ve) Control (BMDM+LPS) (Lysate/Supernatant)
WT
WT
WT
HD
HD
HD
WT
WT
WT
HD
HD
HD
NLRP 3 KO
IL1b KO
NLRP 3+
IL-1β +
Caspase-1 (Shorter exposure)
50 kDa
Pro-caspase-1 (45 kDa)
37 kDa
20 kDa
15 kDa
Cleaved caspase-1 (p10/12)
Caspase-1 (Longer exposure)
50 kDa
Pro-caspase-1 (45 kDa)
37 kDa
20 kDa
15 kDa
Cleaved caspase-1 (p10/12)

Supplementary Figure 7 NLRP3, IL-1β and caspase-1 protein expression in non-treated WT and HD control brain samples (Hippocampus and Striatum). NLRP3 and IL-1β (a), caspase-1 (b). NLRP3, IL-1β and active caspase-1 were below detectable range in brain of HD mice at the baseline by western blot analyses. Positive controls, BMDM: LPS + Nig. Abbreviations: WT, wild type; HD, Huntington's disease, NLR, NLRP3; IL-1β, Interleukin 1 beta; KO, knock out; BMDM, Bone-marrow-derived macrophage; LPS, Lipopolysaccharides; Nig, nigericin. After transferring the protein from gel to membrane, ponceau S staining was performed and then membrane was split into two parts based on the size of the protein of interest. Upper part of the membrane was probed with NLRP3 antibody. While lower part of the membrane was first probed with IL-1β followed by caspase-1. Membrane was stripped using stripping buffer in between two experiments of IL-1β and caspase-1 detection. Representative image of ponceau S staining as loading control for both NLRP3 and IL-1β (a), and caspase-1 (b) is shown in NLRP3 and IL-1β (a) panel only as it was derived from a single membrane and image was captured before probing with any antibody.
